# Production of Vitamin B2 (Riboflavin) by Microorganisms: An Overview

**DOI:** 10.3389/fbioe.2020.570828

**Published:** 2020-11-12

**Authors:** Liudmila A. Averianova, Larissa A. Balabanova, Oksana M. Son, Anna B. Podvolotskaya, Liudmila A. Tekutyeva

**Affiliations:** ^1^Department of Bioeconomy and Food Security, School of Economics and Management, Far Eastern Federal University, Vladivostok, Russia; ^2^Laboratory of Marine Biochemistry, G.B. Elyakov Pacific Institute of Bioorganic Chemistry, Far Eastern Branch, Russian Academy of Sciences, Vladivostok, Russia; ^3^ARNIKA, Territory of PDA Nadezhdinskaya, Primorsky Krai, Russia

**Keywords:** riboflavin, vitamin B2, genetically modified microorganisms, food/feed additive, metabolic engineering

## Abstract

Riboflavin is a crucial micronutrient that is a precursor to coenzymes flavin mononucleotide and flavin adenine dinucleotide, and it is required for biochemical reactions in all living cells. For decades, one of the most important applications of riboflavin has been its global use as an animal and human nutritional supplement. Being well-informed of the latest research on riboflavin production via the fermentation process is necessary for the development of new and improved microbial strains using biotechnology and metabolic engineering techniques to increase vitamin B2 yield. In this review, we describe well-known industrial microbial producers, namely, *Ashbya gossypii*, *Bacillus subtilis*, and *Candida* spp. and summarize their biosynthetic pathway optimizations through genetic and metabolic engineering, combined with random chemical mutagenesis and rational medium components to increase riboflavin production.

## Introduction to Riboflavin

Vitamins are complex organic compounds required in trace amounts for normal functions of an organism. However, mammals cannot produce many vitamins on their own and these have to be externally obtained from dietary supplements and feed additives. Over the last few decades, large-scale production of vitamins by microorganisms has been carried out and more than half of the commercially produced vitamins are fed to domestic animals (Ledesma-Amaro et al., 2015; Lee, 2015).

Riboflavin (vitamin B2) is a water-soluble vitamin, which is produced by all plants and most microorganisms and is essential for growth and reproduction of humans and animals (Revuelta et al., 2016). Riboflavin performs its biochemical function as a precursor for the coenzymes, flavin adenine dinucleotide (FAD) and flavin mononucleotide (FMN), which are mostly involved in redox reactions of all organisms. These flavocoenzymes participate in the metabolism of carbohydrates, lipids, ketone bodies, and proteins from which living organisms derive most of their energy. Additionally, riboflavin promotes the conversion of tryptophan into niacin and vitamins B6 and B9 into their active forms, as well as the mobilization of iron. Therefore, the recommended dietary allowance (RDA) for human and animal nutrition are 0.4–0.6 mg/day and 0–17.5 mg/kg of riboflavin, respectively (Revuelta et al., 2016; Cisternas et al., 2018; EFSA and FEEDAP Panel, 2018).

Industrial production of riboflavin can be performed by both chemical synthesis and fermentation. The fermentation route allows the production of vitamin B2 in a single step, which is cost-effective. In contrast, chemical processes are multistage and expensive. Thus, nowadays, the fermentative production of riboflavin is economically and ecologically more feasible and has completely replaced chemical synthesis. The world market for riboflavin production for human and animal use has more than doubled in 13 years, from 4000 t a^–1^ in 2002 to 9000 t a^–1^ in 2015 (Schwechheimer et al., 2016). Approximately 70% of riboflavin currently available on the market is primarily used as a feed additive, namely Vitamin B2 (80% grade), that is produced via fermentation with genetically modified strains. Global producers, such as BASF (Germany), DSM (formerly Roche; Netherlands), Hubei Guangji Pharmaceuticals, and Shanghai Acebright Pharmaceuticals (formerly Desano; China), derive riboflavin from the cells of industrial microbial strains of *Ashbya gossypii*, *Candida famata* var. *flareri*, and *Bacillus subtilis*, reaching a titer of up to 15, 20, and 14 g/L, respectively (Lim et al., 2001; Revuelta et al., 2016).

Over the last few decades, several groups of researchers have reported successful achievements in the construction of genetically modified strains of species, such as *Escherichia coli, B. subtilis*, *Corynebacterium ammoniagenes*, and *Candida* spp., by applying metabolic engineering strategies. More frequently, such strategies have led to the overexpression of structural and regulatory genes involved in the synthesis of riboflavin or that of its precursors; consequently, this has improved strain productivity and yield of the industrial fermentation product (Perkins et al., 1999a,b; Koizumi et al., 2000; Taniguchi and Wendisch, 2015; Wang et al., 2015). However, there are still unresolved issues caused by various nonspecific reactions in riboflavin biosynthesis, which are not yet completely understood.

The present review summarizes the latest scientific studies that have investigated microorganism-derived riboflavin synthesis using different methods, such as media component optimization, mutations and screening, genetic engineering, and biocatalyst conversion, to improve the production of vitamin B2 and its precursors. The application of these studies is highlighted by references to recent patents related to scientific and industrial developments in microbial riboflavin production.

## Riboflavin-Producing Microorganisms and Culture Conditions

The first commercial microbiological production of riboflavin using bacteria was performed with *Clostridium acetobutylicum* by acetone-butanol fermentation, where riboflavin was formed as a byproduct (Leviton, 1946).

Later, several species of fungi, such as *Eremothecium ashbyii*, *A. gossypii*, *Pichia guilliermondii* (asporogenic *Candida guilliermondii*), *C. famata*, *Candida boidinii*, *Schwanniomyces occidentalis, Pichia caribbica, Candida oleophila, Aspergillus terreus*, and methanol-utilizing *Hansenula polymorpha* were reported as naturally flavinogenic microorganisms capable of synthesizing riboflavin from two major precursors: ribulose 5-phosphate (Ribu5P; pentose phosphate pathway) and guanosine triphosphate (purine pathway) (Sabry et al., 1993; Leathers and Gupta, 1997; Suryadi et al., 2000; Babyak et al., 2002; Abbas and Sibirny, 2011; Ohara et al., 2016). However, these microorganisms accumulated riboflavin slowly and at a low concentration, which were not satisfactory for commercial production of riboflavin.

According to the old Demain classification, microorganisms capable of accumulating more than 10 mg/L of riboflavin were recognized as overproducers and had been subsequently divided into three groups: weak (producing approximately 10 mg/L), moderate (>600 mg/L), and strong (>10 g/L) (Table 1). Since then, numerous experiments have been performed on riboflavin biosynthesis optimization via microbial strain improvement using biological, genetic, and bioinformatics approaches.

**TABLE 1:** Notable riboflavin-producing microorganisms.

Strain and related characteristics	Fermentation media composition and conditions	Yield of riboflavin	Related references
***Bacteria***			
*C. acetobutylicum*, Weizmann strain No. 4259, wild-type	0.5 gm. K_2_HPO_4_, 0.5 gm. MgSO_4_⋅7H_2_O, 0.3 gm. CaCl_2_⋅2H_2_O, 2.0 gm. (NH_4_)_2_SO_4_, 2.0 gm. asparagine, 30.0 gm. lactose, 1.6 × 10^–3^ gm. potassium iodide, 2.75 gm. sodium lactate, 1 × 10^–6^ gm. biotin and 50 × 10^–6^ gm. para-aminobenzoic acid	0.097 g/L	Leviton, 1946
*B. subtilis* PRF93; genetically engineered	Glucose, 3.6 g; KH_2_PO_4_, 4 g; (NH_4_)SO_4_, 2 g; MgSO_4_⋅7H_2_O, 0.2 g; and 10 ml of trace element solution with the following composition (per liter of distilled water): CaCl_2_⋅2H_2_O, 0.55 g; FeCl_3_, 1 g; MnCl_2_⋅4H_2_O, 0.1 g; ZnCl_2_, 0.17 g; CuCl_2_⋅2H_2_O, 0.043 g; CoCl_2_ 6H_2_O, 0.06 g; and Na_2_MoO_4_ 2H_2_O, 0.06 g; pH 6.6, T 37°C, time 45 h	0.08 g/L	Sauer et al., 1996
*B. subtilis* RB50:(pRF69)6o(Ade+) genetically engineered	Glucose, 25 g; yeast extract, 20 g; KH_2_PO_4_, 7.5 g; MgCl_2_⋅H_2_O 1.5 g; CaCl_2_⋅2H_2_O, 1.0 g; MnSO_4_, 0.05 g; FeCl_3_⋅6H_2_O 0.025 g; Mazu DF37C 2.5 g; sodium glutamate, 5 g; (NH_4_)_2_SO_4_, 0.3 g; pH 6.8, T 39°C, time 56 h	4–15 g/L	Perkins et al., 1999b
*B. subtilis* ATCC 6051, wild-type	Fructose, 38.10 g; MgSO_4_, 0.85 g; K_2_HPO_4_, 2.27 g; FeSO_4_, 0.02 g; yeast extract; 4.37 g, T 30°C, time 72 h	3.85 mg/L	Oraei et al., 2018
*B. subtilis* AJ12644, mutated	Glucose, 80 g; NH_4_Cl, 15 g; KH_2_PO_4_, 0.2 g; MgSO_4_⋅7H_2_O, 0.4 g; Fe^2+^, 2 mg; Mn^2+^, 2 mg; RNA, 1.2 g; CaCl_2_⋅2H_2_O, 2 g; Soybean protein hydrolizate, 40 ml; L-glutamic acid, 10 g; L-methionine, 0.3 g; pH 7.5 during fermentation, T 34°C, time 16 h	1.05 g/L	Usui et al., 1997
*B. subtilis* KCCM 10445, mutated	Glucose, 100 g; dry yeast, 20 g; corn steep liquor, 5 g; magnesium sulfate 7-hydrate, 0.5 g; monopotassium phosphate, 1.5 g; dipotassium phosphate, 3.5 g; urea, 6 g; erythromycin, 10 mg; chloramphenicol, 10 mg; pH 7.2–7.4, T 37°C, time 90 h	26.8 g/L	Lee et al., 2004b
*B. subtilis* KCCM 10446, mutated	Dry yeast, 20 g; corn steep liquor, 5 g; magnesium sulfate 7-hydrate, 0.5 g; monopotassium phosphate, 17.5 g; dipotassium phosphate, 7.5 g; ammonium sulfate, 2 g supplement medium: 620 g/l of glucose, 26.7 g/l of dry yeast, 26.7 g/l of corn steep liquor; pH 7.2–7.4, T 37°C, time 60–70 h	26.5 g/L	Lee et al., 2004a
*B. subtilis* VKPM-B 6797 genetically engineered	Molasses, 15 g; yeast extract, 1.5 g; (NH_4_)_2_HPO_4_, 14.2 g; Ê_2_SO_4_, 5.33 g; MgSO_4_⋅7H_2_O, 0.71 g; pH 6.5–7.2, T 37–41°C, time 42 h	12.4 g/L	Debabov et al., 1997
*B. subtilis* RH44, genetically engineered	Glucose, 80 g; yeast extract, 5 g; K_2_HPO_4_, 1 g; H_2_PO_4_, 1 g; MgSO_4_⋅7H_2_O, 1 g; pH 7.2, T 41°C, time 48 h	16.36 g/L	Wu et al., 2007
*B. subtilis* X42, genetically engineered	Yeast powder, 20.8 g; glucose, 100 g; urea, 4.8 g; CuCl_2_, 0.024; MgSO_4_⋅7H_2_O, 0.5 g; KH_2_PO_4_, 1 g; FeCl_2_, 0.02 g	7.9 g/L	Li et al., 2013
*B. subtilis* RF1, genetically engineered	Glucose⋅H_2_O, 600 g; yeast extract, 10 g; (NH_4_)_2_HPO_4_, 6 g; KH_2_PO_4_, 5 g; MgSO_4_⋅7H_2_O, 0.5 g; pH 6.9, T 40°C, time 48 h	9.4 g/L	Man et al., 2014
*C. ammoniagenes*, genetically engineered (plasmid pFM76)	Corn steep liquor, 20 g; glucose, 150 g; KH_2_PO_4_, 10 g; K_2_HPO_4_, 10 g; MgSO_4_⋅7H_2_O, 10 g; CaCl_2_⋅2H_2_O, 100 mg; FeSO_4_⋅7H_2_O, 10 mg, ZnSO_4_⋅7H_2_O, 5 mg; MnSO_4_⋅H_2_O, 2 mg; CuSO_4_⋅5H_2_O, 0.5 mg; L-cysteine-HCl, 20 mg; thiamine-HCl, 5 mg; calcium pantothenate, 10 mg; nicotinic acid, 20 mg; biotin, 0.09 mg; adenine, 200 mg; urea, 2 g; spectinomycin, 100 mg; pH 7.5, T 32°C, time 72 h	15.3 g/L	Koizumi et al., 2000
*Lactococcus lactis* subsp. *cremoris* NZ9000, (pNZGBAH), genetically engineered	M17 medium supplemented with 0.5% glucose (GM17); T 37°C, time 3 h	24 mg/L	Burgess et al., 2004
*Lactobacillus fermentum* KTLF1	MRS medium: peptone, 10 g; beef extract, 10 g; yeast extract, 5 g; tween 80, 1 g; glucose, 20 g; sodium acetate, 5 g; ammonium citrate, 2 g; K_2_HPO_4_, 2 g; magnesium sulfate, 0.2 g; manganese sulfate, 0.05 g; pH 6.5, T 37°C	2.36 mg/L	Thakur et al., 2015
*Corynebacterium glutamicum* KCCM11223P, genetically engineered	Glucose, 1 g; (NH_4_)_2_HPO_4_, 20.0 g; soybean extract, 2.5 g; corn steep solids, 5 g; urea, 3 g; KH_2_PO_4_, 1 g; MgSO_4_⋅7H_2_O, 0.5 g; biotin, 100 μg; thiamine HCL, 1000 μg; calcium pantothenic acid, 2000 μg; nicotinamide, 3000 μg; CaCO_3_, 30 g; pH 7.0, T 30°C, time 48 h	245 mg/L	Park et al., 2014
*E. coli* RF05S-M40, genetically engineered	Na_2_HPO_4_, 3.8 g; KH_2_PO_4_, 1.5 g; (NH_4_)_2_SO_4_, 1.0 g; MgSO_4_, 0.2 g; yeast extract, 5 g; T 31°C, time 48 h	2702.8 mg/L	Lin et al., 2014
***Fungi***			
*A. gossypii* AgOXA50, wild-type	Corn steep liquor, 60 g; gelatin, 30 g; KH_2_PO_4_, 1.5 g; glycine, 1.5 g, Co^2+^, 2 μg; Mn^2+^, 5 μg; Zn^2+^, 10 μg; Mg^2+^, 1 μg, rapeseed oil, 50 g; pH 6.8, T 28°C, time 8 days	5.2 g/L	Sugimoto et al., 2010
*A. gossypii*, wild-type	Soybean oil, bone fat, corn steep liquor, gelatin	5.0 g/L	Szczesniak et al., 1971
*A. gossypii* ZP4, mutated	Corn steep liquor, 60 g; gelatin, 30 g; KH_2_PO_4_, 1.5 g; glycine, 1.5 g, Co^2+^, 2 μg; Mn^2+^, 5 μg; Zn^2+^, 10 μg; Mg^2+^, 1 μg, rapeseed oil, 50 g; pH 6.8, T 28°C, time 5 days	8.7 g/L	Park et al., 2007
*A. gossypii* NRRL Y-1056, wild-type	Whey; whey with different supplements: bran, soybean flour, glycine and peptone, sucrose, glycine, yeast extract, peptone, and soybean oil; pH 5.1, T 28°C, time 8 days	0.03 g/L	Ertrk et al., 1998
*A. gossypii* W 122032, genetically engineered	Corn steep liquor, 60 g; gelatin, 30 g; KH_2_PO_4_, 1.5 g; glycine, 1.5 g, Co^2+^, 2 μg; Mn^2+^, 5 μg; Zn^2+^, 10 μg; Mg^2+^, 1 μg, rapeseed oil, 73 g; pH 6.8, T 28°C, time 9 days	13.7 g/L	Park et al., 2011
*A. gossypii* ATCC 10895, wild-type	Gelatin, 30 g; corn steep liquor, 60 g; glycine, 1.5 g; KH_2_PO_4_, 1.5 g; CoCl_2_⋅6H_2_O_2_ mg; MnCl_2_⋅4H_2_O, 5 mg; ZnSO_4_⋅7H_2_O, 10 mg; MgS_2_O_3_⋅6H_2_O, 1 mg; soybean oil, 50 g; pH 6.8, T 28°C, Time 4 days	2.5 g/L	Lim et al., 2003
*E. ashbyii*, wild-type	Glucose, 50 g; peptone, 30 g; KH_2_PO_4_, 2 g; MgSO_4_, 1 g; NaCl, 1 g; yeast extract, 60 g; pH 6.5–7.0, T 28°C, time 144 h	1.5–3.1 g/L	Debabov et al., 1997
*E. ashbyii* NRRL 1363, wild-type	Molasses, 50 g; peanut seed cake, 50 g; KH_2_PO_4_, 2 g; MgSO_4_, 0.1 g; NaCl, 1 g; 80 tween, 1.8 ml; pH 6.5, T 30°C, time 6 days	2.85 g/L	Kalingan and Liao, 2002
*A. terreus*, wild-type	Centrifuged beet-molasses, 90 g; L-asparagine, 1 g; MgSO_4_⋅7H_2_O, 0.5 g; K_2_HPO_4_/KH_2_PO_4_ (1:1), 5 g; pH 8.0, T 30°C, time 16 days	1,0 g/L	Sabry et al., 1993
***Yeast***			
*C. famata* ATCC 20849; 20850, mutated	Yeast extract, 3 g; malt extract, 3 g; peptone, 5 g; carbon source (2%) 20 g + mineral supplements; pH 7.0, T 30°C, time 200 h	20 g/L	Heefner et al., 1992
*C. famata* ATCC 20755; 20756, mutated	yeast extract, 3 g; malt extract, 3 g; peptone, 5 g; carbon source (2%) 20 g + mineral supplements; pH 7.0, T 30°C, time 6 days	7.5 g/L	Heefner et al., 1993
*Candida* sp. LEB 130, wild-type	Sucrose, 30 g; KH_2_PO_4_, 2 g; MgSO_4_, 1 g; ZnSO_4_, 0.5 mL; pH 7.0, T 30°C, time 48 h	12.5 μg/mL	Suzuki et al., 2011
*P. guilliermondii* NRRL Y-488, wild-type	Xylose/glucose, 2%; yeast extract, 1%; yeast nitrogen base, 0.67%; time 48 h	3.1–4.5 μg/mL	Leathers and Gupta, 1997
*P. guilliermondii* XS-3 genetically engineered	Burkholder medium supplemented; T 30°C, time 50 h	0.003 g/L	Babyak et al., 2002
*P. guilliermondii* DM 644, wild-type	Oil substrate, 10 g; urea, 2.5 g; pH 5.0, T 30°C, time 24 h	19.12 μg/mL	Pessoa et al., 2003
*Pichia pastoris* X-33 ScRIB1, genetically engineered	Glucose⋅1H_2_O, 550 g, KCl, 10 g; MgSO_4_⋅7H_2_O, 6.45 g, CaCl_2_⋅2H_2_O, 0.35 g, and 12 ml PTM trace salts stock solution (per liter): 6.0 g CuSO_4_⋅5H_2_O, 0.08 g NaI, 3.0 g, MnSO_4_⋅H_2_O, 0.2 g Na_2_MoO_4_⋅2H_2_O, 0.02 g H_3_BO_3_, 0.5 g, CoCl_2_, 20.0 g ZnCl_2_, 65.0 g FeSO_4_⋅7H_2_O, 0.2 g biotin, 5.0 ml H_2_SO_4_ (95–98%); pH 5.0, T 25°C, time 24–50 h	0.175 g/L	Marx et al., 2008
*S. cerevisiae* NH-268, mutated	Calcium acetate 132 g; (NH_4_)_2_SO_4_, 6 g; KH_2_PO_4_, 1 g; MgSO_4_⋅7H_2_O, 2 g; ZnSO_4_⋅7H_2_O, 11 mg; pH 7.0, T 30°C, time 11 days	3.4 g/L	Matsuyama et al., 1987
*S. cerevisiae*, mutated	Calcium acetate 103 g; (NH_4_)_2_SO_4_, 3 g; KH_2_PO_4_, 2 g; MgSO_4_⋅7H_2_O, 1 g; ZnSO_4_⋅7H_2_O 2.2 mg; pH 7.0, T 30°C, time 4 days	0.5–2.5 g/L	Kimitoshi et al., 1988

*ATCC, American Type Culture Collection, United States; KCCM, Korean Culture Center of Microorganisms, South Korea; NRRL, Northern Regional Research Laboratory also known as the Agricultural Research Service (ARS) Culture Collection, United States; CBS-KNAW Collections, Netherlands.*

The fermentative production of riboflavin is naturally carried out by the wild-type flavinogenic ascomycetes, such as *E. ashbyii* and *A. gossypii*, with the accumulation of riboflavin in mycelia at the end of the growth phase, which provides the fungi with a bright yellow color (Aguiar et al., 2015). Among them, *A. gossypii* is commercially preferred as it maintains a steady high-producing capacity of riboflavin, whereas highly flavinogenic clones of *E. ashbyii* easily lose their potential during lyophilization or storage at room temperature, resulting in their genetic instability and low productivity (Abbas and Sibirny, 2011). However, *E. ashbyii* is able to overproduce FAD, unlike *A. gossypii* (Kalingan and Liao, 2002). Modern approaches guided by genetic manipulations and medium supplementation have led to riboflavin overproduction in these organisms (Table 1). Althofer et al. (2005) described increases in the riboflavin yields of up to 135% (3.8 g/L) in *A. gossypii* compared to the unmodified ATCC 10895 strain (Table 1). Increased riboflavin production was shown for the *A. gossypii* wild-type by supplementation of glycine and hypoxanthine, which are precursors for GTP (Monschau et al., 1998; Table 1). Park et al. (2007) improved riboflavin yield threefold, using *A. gossypii* spores that were mutated by UV light exposure. The addition of activated bleaching earth containing 75 g/L rapeseed oil and oxygen-enriched air to the mutated strain ZP4 culture increased riboflavin concentration to 8.7 g/L after 5 days cultivation (Park et al., 2007; Table 1). Using genetic techniques and supplement optimization, *A. gossypii* strains could yield as high as 13.7 g/L of riboflavin (Park et al., 2011; Table 1).

Among *Candida* strains, the mutant *C. famata* ATCC 20849 demonstrates the highest flavinogenic potential, but its extreme sensitivity to the presence of iron makes the fermentation process complicated (Heefner et al., 1992, 1993; Table 1). The maximum amounts of riboflavin produced by the yeast *C. famata* under conditions of iron deficiency were between 560 mg/L and 7.5 g/L (Heefner et al., 1993). Additionally, riboflavin production of up to 20 g/L in 200 h was reported from *C. famata* and its mutants obtained by selection for resistance to 2-deoxyglucose (DOG), iron, tubercidin (a purine analog), and depleted medium (Heefner et al., 1992; Table 1). As the biosynthesis process depends on the addition of nitrogen sources, such as glycine and hypoxanthine, selection for strains resistant to the adenine antimetabolite, 4-aminopyrazolo (3, 4-d) pyrimidine, improved production (Park et al., 2007, 2011; Sugimoto et al., 2010; Table 1). Threonine demonstrated a ninefold stimulation in a strain with a cloned threonine aldolase gene, responsible for converting threonine to glycine (Abbas and Sibirny, 2011).

Among other fungi, the filamentous *Aspergillus niger*, *A. terreus, Aspergillus flavus*, *Penicillium chrysogenum*, and *Fusarium* have also been reported as flavin producers (Abbas and Sibirny, 2011). The Japanese inventors have developed a riboflavin production process using non-flavinogenic yeast *Saccharomyces cerevisiae* grown in the presence of calcium acetate and zinc ions. The productivity of this process was up to 3.4 g/L riboflavin without impurity problems, compared to the molasses-grown cells (Matsuyama et al., 1987; Table 1). Some flavogenic yeast mutants of *P. guilliermondii*, especially those capable of riboflavin uptake and accumulation, can also be employed in biotechnology (Babyak et al., 2002; Table 1). The recombinant strain XS-3 produced three times more riboflavin (3.6 mg/L) compared to the wild-type strain ATCC 9058/L2 (1.2 mg/L) under the conditions described. Daneshazari et al. (2013) reported that yeasts, *Rhodosporidium diobovatum* and *Trichosporon asahii*, are also able to produce riboflavin (Daneshazari et al., 2013).

However, riboflavin biosynthesis has been most studied on the nonpathogenic bacterium, *B. subtilis*, which has become a model organism among industrial riboflavin-producing strains due to its ability to secrete large amounts of protein directly into the medium in a short time (Sauer et al., 1996; Perkins et al., 1999a,b; Lee et al., 2004a,b; Wu et al., 2007).

*Bacillus subtilis* is capable of producing riboflavin precursors, inosine and guanosine, in the purine pathway, which could be converted metabolically into riboflavin. However, riboflavin overproduction has been achieved by obtaining mutants with overexpression of certain genes and resistance to purine analogs azaguanine, decoyinine, and methionine sulfoxide, or the riboflavin analog roseoflavin, as the *B. subtilis* riboflavin pathway was found to be carried through genes organized in the *rib* operon (Debabov et al., 1997; Perkins et al., 1999a,b; Lee et al., 2004a,b; Paracchini et al., 2017; Table 1).

Strains *B. subtilis* KU559874 and *Bacillus tequilensis* KU559876 demonstrated high potentiality for riboflavin production (Table 1). The addition of glycine into their nutrition medium was effective, and the influencing concentration was 1 g/L, allowing for riboflavin yields of 144.7 and 184.2 mg/L, respectively (Abd-Alla et al., 2016). *B. subtilis* VKPM-B 6797, harboring plasmid 62/pMX30ribO186, and producing up to 12.5 g/L of riboflavin in a 42 h fermentation, was developed by Debabov et al. (1997). The strain VKPM-B 6797 was obtained from the *B. subtilis* mutant RK6121 resistant to 8-azaguanine, methionine sulfoxide, diacetyl, and psicofuranine which contained, in addition, a plasmid with the mutated *rib* operon (Table 1). Oraei et al. (2018) studied optimal concentrations of 13 minerals during *B. subtili*s ATCC 6051 fermentation to enhance riboflavin production on a fructose substrate (Table 1). The results revealed that concentrations of MgSO_4_, K_2_HPO_4_, and FeSO_4_ had greater influence on riboflavin production (3.85 mg/L) (Oraei et al., 2018). Li et al. (2013) screened 11 medium components for riboflavin production of recombinant *B. subtilis* X42 by metabolic design. Among tested variables, glucose, yeast powder, MgSO_4_, urea, CuCl_2_, and MnCl_2_ had the greatest effects on riboflavin production (Table 1). Wu et al. (2007) increased levels of riboflavin in *B. subtilis* RH44 up to 16.4 g/L in 48 h with optimum medium components obtained by statistical experimental design (Table 1). Among 15 variables, glucose, NaNO_3_, K_2_HPO_4_, ZnSO_4_, and MnCl_2_ were identified as the most crucial factors for riboflavin production (Wu et al., 2007).

Recently, the availability of advanced genetic engineering technology, combined with process development and optimization, could allow certain bacteria such as *Salmonella typhimurium*, *C. ammoniagenes, Corynebacterium glutamicum*, *E. coli*, which are not natural overproducers, to become attractive microorganisms for riboflavin biosynthesis research (Koizumi et al., 2000; Park et al., 2014; Taniguchi and Wendisch, 2015; Wang et al., 2015). *Mycobacterium phlei* was able to produce small quantities of riboflavin from beet molasses (Abd-Alla et al., 2016). *C. ammoniagenes* was used for the industrial production of purine and pyrimidine nucleotides and was thus selected for developing an alternative bacterial riboflavin producer. Under optimized conditions, the engineered strain accumulated 15.3 g/L of riboflavin in 72 h, which is comparable to the *B. subtilis* yield (Koizumi et al., 2000; Table 1). Succinate-utilizing *Rhizobium* sp. was shown to produce riboflavin, as well as other B-group vitamins (Sierra et al., 1999).

The use of lactic acid bacteria (LAB) is a common practice in the dairy industry, and the addition of riboflavin-producing strains to fermented products, such as fermented milk, yogurt, and cheese, increases riboflavin concentrations, which is economically viable. Recent study on riboflavin biosynthesis during food fermentation in dairy products showed that fermentation of cow milk with *Lactococcus lactis* and *Propionibacterium freudenreichii* ssp. *shermanii* as starter cultures significantly increased the riboflavin content of milk (Le Blanc et al., 2011). Burgess et al. (2004, 2006) characterized riboflavin synthesis in *L. lactis* subsp. *cremoris* NZ9000, which can be used as a model for strain design for essential vitamin production (Table 1). Thakur et al. (2015) reported riboflavin production in *Lactobacillus fermentum* KTLF1 (2.36 mg/L) and *Lactobacillus plantarum* (2.13 mg/L) (Table 1). According to Jayashree et al. (2010), the efficient riboflavin-producing strain *L. fermentum* MTCC 8711 showed 2.29 mg/L of riboflavin in MRS broth after 24 h (Jayashree et al., 2010). Guru and Viswanathan (2013) reported that *Lactobacillus acidophilus* produces higher riboflavin levels compared with *L. lactis* on whey substrate (Guru and Viswanathan, 2013). Sybesma et al. (2004) developed the *L. lactis* strain, using direct mutagenesis and metabolic engineering for simultaneous overproduction of both folate and riboflavin (Sybesma et al., 2004). Thus, LAB are attractive riboflavin producers having the potency to extend their biosynthetic capacity by modern biotechnology methods (Thakur et al., 2015).

Presently, two major overproducers of commercial riboflavin include the yeast-like mold, *A. gossypii*, which synthesizes riboflavin in concentrations greater than 13 g/L, and recombinant *B. subtilis* strains that produce at least 26.8 g/L riboflavin (Lee et al., 2004b; Park et al., 2011; Table 1).

## Biosynthesis of Riboflavin and Its Derivatives

Riboflavin biosynthesis begins from two major substrates, GTP and Ribu5P, derived from purine biosynthesis or/and the pentose phosphate pathway, containing seven enzymatic steps generating the final product (Liu et al., 2020). Research on riboflavin biosynthesis demonstrated that characteristic features of most enzymes and steps involved in the riboflavin pathway are mostly similar between prokaryotes and plants, whereas fungi use a somewhat different pathway and enzymes (Abbas and Sibirny, 2011). To produce GTP and Ribu5P precursors, industrial microorganisms *C. famata* and *B. subtilis* utilize glucose, whereas *A. gossypii* prefers fatty acids.

**FIGURE 1:**
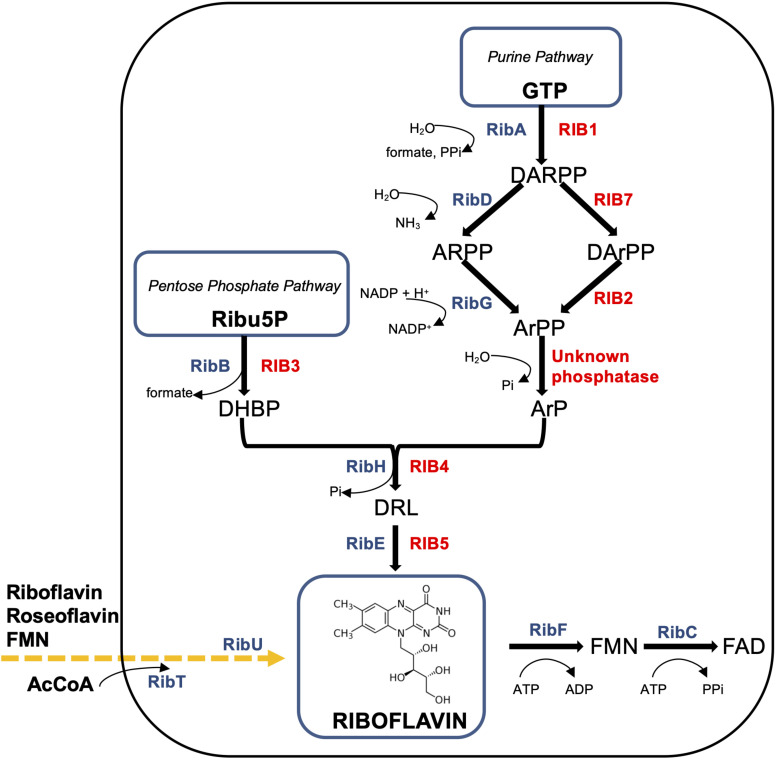
Schematic diagram of the riboflavin pathways in *Ashbya gossypii* and *Bacillus subtilis. B. subtilis* enzymes (in blue color): RibAB, bifunctional enzyme GTP cyclohydrolase II (A)/3,4-DHBP synthase (B); RibDG, bifunctional deaminase (D)/reductase (G); RibH, lumazine synthase; RibE, riboflavin synthase; RibFC, bifunctional flavokinase (F)/FAD synthetase (C). The phosphatase converting ArPP to ArP is unknown. RibU (originally known as ypaA) – riboflavin transmembrane transporter, its substrates are FMN and riboflavin analogue roseoflavin; RibT, N-acetyltransferase GCN5 (earlier predicted as transporter), which transfers the acetyl group from acetyl coenzyme A (AcCoA) to a variety of substrates (unknown). Produced or consumed cofactors are shown with curved arrows near the Rib enzymes. *A. gossypii* enzymes (in red color): RIB1, GTP cyclohydrolase II; RIB7, DARPP reductase; RIB2, DArPP deaminase; RIB3, DHBP synthase; RIB4, lumazine synthase; RIB5, riboflavin synthase. Precursors (metabolites): GTP, guanosine triphosphate; DARPP, 2,5-diamino-6-ribosyl-amino-4(3H)pyrimidinedione 5′-phosphate; ARPP, 5-amino-6-ribosyl-amino-2,4(1H,3H)pyrimidinedione 5′-phosphate; DArPP, 2,5-diamino-6-ribityl-amino-4(3H)pyrimidinedione 5′-phosphate; ArPP, 5-amino-6-ribityl-amino-2,4(1H,3H)pyrimidinedione 5′-phosphate; ArP, 5-amino-6-ribityl-amino-2,4(1H,3H)pyrimidinedione; DHBP, 3,4-dihydroxy-2-butanone-4-phosphate; DRL, 6,7-dimethyl-8-ribityllumazine; Ribu5P, ribulose 5-phosphate; FMN, flavin mononucleotide; FAD, flavin adenine dinucleotide.

Most knowledge on riboflavin biosynthesis today has been obtained in considerable detail for two major industrial producers: the filamentous fungus *A. gossypii* and the Gram-positive bacterium *B. subtilis* (Figure 1).

In *B. subtilis*, the biosynthetic pathway carried out by the *rib* operon consists of five genes – *ribDG, ribE, ribAB, ribH, ribT* (Pedrolli et al., 2015). The genome of *A. gossypii* is organized into seven chromosomes and genes responsible for riboflavin biosynthesis, and it is not clustered as in bacteria. The six riboflavin biosynthetic genes encoding riboflavin enzymes in *A. gossypii*, *RIB1, RIB2, RIB3, RIB4, RIB5, RIB7*, and their regulation are highly similar to those of *S. cerevisiae*, which has become a popular model for fungal development biology (Ledesma-Amaro et al., 2014; Aguiar et al., 2015).

The first step of the purine pathway for riboflavin biosynthesis begins in a similar manner in all microorganisms, from the conversion of GTP into 2,5-diamino-6-ribosyl-amino-4(3H)pyrimidinedione 5′-phosphate (DARPP), formate, and pyrophosphate catalyzed by GTP cyclohydrolase II (Liu et al., 2020). The gene *RIB1* in *A. gossypii* and hybrid bifunctional *ribAB* in *B. subtilis* encode this enzyme (Figure 1). The overexpression of *ribAB* in *B. subtilis* resulted in 25% greater riboflavin, indicating the biosynthesis rate-limiting step (Hümbelin et al., 1999).

Thereafter, DARPP is converted into 5-amino-6-ribityl-amino-2,4(1H,3H)pyrimidinedione (ArP) by sequential reactions of deamination, side chain reduction, and dephosphorylation (Figure 1). In *A. gossypii*, DARPP is exposed by a reduction reaction and a subsequent deamination by corresponding enzymes DARPP reductase (encoded by *RIB7*) and DArPP deaminase (encoded by *RIB2*), to generate 5-amino-6-ribityl-amino-2,4(1H,3H)pyrimidinedione 5′-phosphate (ArPP) (Figure 1). Notably, in *B. subtilis*, as in plants, the deamination occurs prior to the reduction, which is in the reverse order and is catalyzed by a bifunctional enzyme encoded by *ribDG* (Pedrolli et al., 2015; Revuelta et al., 2016).

The next step might be the dephosphorylation of ArPP (Figure 1). However, the dephosphorylation mechanism as well as phosphatase that catalyzes conversion of ArPP into ArP remains to be elucidated in the riboflavin biosynthesis pathway, though much investigative work has been performed on the origin of the four carbons of ArP. A specific phosphatase, catalyzing ArPP dephosphorylation, has been found in *planta* Arabidopsis among eight enzymes from the haloacid dehydrogenase (HAD) superfamily, whereas the search for similar enzymes with promiscuous functions in *B. subtilis* was not successful in determining a candidate for that role (Sa et al., 2016).

The alternative pentose phosphate pathway of riboflavin biosynthesis includes the catalytic conversion of Ribu5P into 3,4-dihydroxy-2-butanone-4-phosphate (DHBP) by DHBP synthase. The encoding genes of this stage are *RIB3* and *ribAB* for *A. gossypii* and *B. subtilis*, respectively (Figure 1). It is important to note that the product of *ribAB* in *B. subtilis* is a fused bifunctional enzyme with GTP cyclohydrolase II and 3,4-DHBP synthase activities, which catalyzes the cleavage of GTP and converts DHBP from Ribu5P in the initial steps of both branches of riboflavin biosynthesis (Figure 1).

Thereafter, both branches of the riboflavin biosynthetic pathway merge into one (Figure 1). Following condensation of ArP with DHBP, yields 6,7-dimethyl-8-ribityllumazine (DRL) catalyzed by DRL synthase (lumazine synthase). The enzyme is encoded by *RIB4* in *A. gossypii* and by *ribH (beta subunit)* in *B. subtilis*, forming with the *ribE* product *(alpha subunit, light enzyme)* the posttranslation luminase/riboflavin synthase complex [(RibE)_3_][(RibH)_60_] (Schwechheimer et al., 2016; Han and Woycechowsky, 2017).

The last step is dismutation of DRL by the riboflavin synthase translated from *ribE* in *B. subtilis* and *RIB5* in *A. gossypii* to form riboflavin and ArP, which is recycled in the riboflavin biosynthetic pathway (Figure 1).

Finally, the bifunctional flavokinaze/FAD-synthase encoded by the *Bacillus* gene, *ribFC*, catalyzes the conversion of riboflavin to FMN and FAD involved in oxidation-reduction reactions at all cellular levels (Revuelta et al., 2016; García-Angulo, 2017; Figure 1). For *A. gossypii* and yeasts, FMN is synthesized by riboflavin kinase encoded by the *FMN1* gene, which could be the major rate-limiting step for FAD provided by the *FAD1* gene product, FAD-synthase (Liu et al., 2020; Patel and Chandra, 2020).

## Riboflavin Biosynthesis Regulation

A variety of inducers, effectors, inhibitors, and signal molecules affect metabolite overproduction in microorganisms that provide positive or negative regulation of enzyme catalyzing metabolic reactions, through regulatory genes responsible for feedback inhibition (transcription and translation levels) or allosteric effects on some enzymes (posttranslational level) (Sanchez and Demain, 2008). Regulation of the riboflavin biosynthetic pathway is not completely understood for several riboflavin-producing microorganisms. However, most studies have unraveled regulatory mechanisms behind riboflavin overproduction linked to nutritional and oxidative stress in microorganisms (Schlosser et al., 2007; Aguiar et al., 2015). As is well known, wild-type microorganisms possess metabolic regulatory systems to prevent an overproduction of riboflavin. The regulation of riboflavin synthesis occurs mostly at the level of its very slow biosynthetic enzymes; thus, it is necessary to induce a strong and stable expression of their encoding genes, which is achieved by stress response, nutrition, or pathway regulation at a certain phase of microbial growth (Acevedo-Rocha et al., 2019).

Schlosser et al. (2007) found that regulation of the three genes *RIB3, RIB4*, and *RIB5* in *A. gossypii* involved in the pentose phosphate pathway branch were regulated upon cessation of growth or oxidative stress due to nutrient depletion and entry into the riboflavin production phase, whereas *RIB2* and *RIB7* belonging to the GTP branch remained constant (Schlosser et al., 2007). A more recent study reported that there was no significant increase at the transcriptional level for all *RIB* genes except *RIB4* during the riboflavin biosynthetic phase (Ledesma-Amaro et al., 2015). In addition, the flavinogenic activity of *A. gossypii* depends on the cultivation temperature, dropping markedly at 38°C. It has been suggested that at elevated temperatures a specific repressor of riboflavin biosynthesis is activated, although no direct evidence has been presented. In *E. ashbyii*, the shift from growth to the production phase was accompanied by depression of GTP cyclohydrolase II and FAD synthetase, whereas the activity of riboflavin synthase was only marginally changed (Abbas and Sibirny, 2011).

The interaction of endogenous riboflavin with light induces oxidative DNA damage in cells by emerging reactive oxygen species (ROS), but exogenous riboflavin was shown to protect *A. gossypii* spores against UV light (Silva et al., 2019; Sugimoto et al., 2010). An *A. gossypii* mutant without sporulation was characterized by lowered riboflavin secretion, and cyclic adenosine monophosphate (cAMP) inhibited both sporulation and riboflavin oversynthesis. It is probable that riboflavin protects spores of fungi and attracts insects to their dispersal (Aguiar et al., 2015). With the induction of riboflavin secretion, enzyme activity involved in detoxification of ROS, e.g., catalase and superoxide dismutase are also induced (Walther and Wendland, 2012; Kavitha and Chandra, 2014). However, Silva et al. (2019) assessed putative genotoxic effects associated with *A. gossypii* riboflavin overproduction and determined that exposure of overproducing cells to sunlight—mimicking light during growth—induced intracellular ROS and DNA damage accumulation together with a 1.5-fold increase in riboflavin production.

The overproduction of riboflavin by *A. gossypii* can be induced by environmental stress, e.g., nutritional or oxidative stress, via the Yap-protein family, which has a well-documented role in stress response. In yeasts, Yap1 absence renders cells hypersensitive to oxidants generated by superoxide anion radicals. Genome expression is operated by Yap1-8 transcription factors, which have the ability to act as both inducers and repressors. Studies on different Yap factors in *S. cerevisiae* are shown to be involved in various stress responses: Yap2/Cad1 is activated in the presence of cadmium, Yap4/Cin5 and Yap6 under osmotic shock, Yap5 under iron overload, and Yap8/Arr1 by arsenic compounds. Yap3 and Yap7 seem to be involved in hydroquinone and nitrosative stresses, respectively (Silva et al., 2015; Rodrigues-Pousada et al., 2019).

Positive effectors of regulation are also iron ions, and occasionally other metals (cobalt, chromium, zinc, magnesium) (Abbas and Sibirny, 2011; García-Angulo, 2017). Flavinogenic yeasts and bacteria have strains that overproduce riboflavin under iron-restrictive conditions, probably due to either the direct role of riboflavin as an electron donor for iron reduction or as a cofactor for enzyme activity. The maximum amount of riboflavin produced by the yeast *C. famata* under conditions of iron deficiency was 560 mg/L (Abbas and Sibirny, 2011). Its mutant was defective for riboflavin oversynthesis in the iron-deficient medium due to the mutated transcription factor gene *SEF1*. Similar data on an iron-deficient growth medium were obtained with mutant *P. guilliermondii* rib83, which was incapable of overproducing riboflavin. It was hypothesized that riboflavin might be involved in the nonenzymatic reduction of weakly soluble Fe^3+^ to Fe^2+^ due to the use of mechanisms for iron assimilation, distinguished from most flavinogenic yeasts that do not overproduce riboflavin under conditions of iron limitation (Dmytruk and Sibirny, 2012). Several enzymes that catalyze biological electron transfer utilize diverse vitamins and/or metals as cofactors. Riboflavin and iron are the primary cofactors, each one assisting approximately 17% of cofactor-requiring enzymes. In bacteria, the transcriptional ferric uptake regulator Fur is the main regulator of iron homeostasis (Cisternas et al., 2018). Research conducted by Vasileva et al. (2012) showed that both iron starvation and Fur deletion highly increased the transcription of the *rib* operon responsible for riboflavin biosynthesis in *C. acetobutylicum*. In *B. subtilis*, Fur-regulated flavodoxins YkuNOP are expressed upon iron starvation and are also likely to replace another Fur-regulated enzyme involved in energy metabolism, ferredoxin. Iron starvation was shown to induce secretion of riboflavin in *Methylocystis* sp., a methanotrophic bacterium (Vasileva et al., 2012).

Co^2+^ caused strong stimulation of riboflavin synthesis in flavinogenic *Candida* species and some stimulation was observed with Cr^6+^, Mn^2+^, and Zn^2+^. In the fungus *A. niger*, riboflavin synthesis was stimulated by a deficiency in Mg^2+^. Some species of flavinogenic yeasts overproduce riboflavin in iron-sufficient media containing n-alkanes as the sole carbon source, but mechanisms of these stimulatory effects remain unknown (Sugimoto et al., 2010; Abbas and Sibirny, 2011; Cisternas et al., 2018).

The riboflavin yield is also markedly dependent on the type and initial concentrations of carbon and nitrogen sources, as well as supplementation of primary or intermediate precursors for biosynthesis. Several studies were conducted on enhancing riboflavin production by supplementation. To produce precursors for GTP and Ribu5P in riboflavin biosynthetic pathways (Figure 1), *B. subtilis* and *A. gossypii* utilize glucose and other sugars as a carbon source. However, for overproduction, *A. gossypii* prefers the use of plant oils (corn or soybean), which are obtained as decomposing fatty acids and glycerol by extracellular lipases. In *A. gossypii*, fatty acids are oxidized into acetyl-CoA (ß-oxidation pathway), then converted into malate through the glyoxylate shunt to enter gluconeogenesis and serve together with the immediate precursor GTP as carbon donors for riboflavin (Schwechheimer et al., 2018). Industrial waste materials, such as oil discharged by oil refinery plants, grape-must, beet molasses, peanut seed cake, and whey, have also been employed in riboflavin production but with limited success. However, researchers are hopeful about riboflavin biosynthesis in *A. gossypii* with glucose, fructose, sucrose, starch, maltose, and degraded collagen as carbon sources (Ledesma-Amaro et al., 2015; Aguiar et al., 2017). As an appropriate supply of carbon source stimulating riboflavin production, *B. subtilis* and *C. famata* can utilize sucrose or maltose instead of glucose (Table 1). Ribitol, purines, or glycine were found to be inductors of riboflavin-producing enzymes at early stage riboflavin biosynthesis with glycine as a preferred limiting precursor of GTP (Schlüpen et al., 2003; Revuelta et al., 2016). Supplementation of glycine during fermentation with *A. gossypii* and *Candida* sp. was not associated with cell growth and doubled riboflavin production without growth variations (Ledesma-Amaro et al., 2014, 2015; Revuelta et al., 2016). Notably, *E. ashbyii* was not stimulated by exogenous glycine, though serine and threonine served as precursors for glycine synthesis (Lim et al., 2001). However, feedback inhibition of important enzymes in their biosynthetic pathways and toxic effects from their excess inhibited cell growth (Lim et al., 2001; Revuelta et al., 2016). During the improvement of riboflavin production by Sugimoto et al. (2010) via optimization of cultural conditions for *A. gossypii*, purines (hypoxanthine) and glycine were critical nutrients for increasing the production by three- to fourfold (Table 1). Xanthine was suggested as an intermediate precursor because of purine structure similarities. However, in experiments with guanine auxotrophs *Aerobacter aerogenes*, *C. guilliermondii*, and *Corynebacterium* sp., lacking xanthine monophosphate (XMP) aminase, it was proven that the main precursor was guanine or a guanine nucleotide and the conversion of adenine, hypoxanthine, and xanthine to riboflavin passed through one of them (Abbas and Sibirny, 2011). Evidently, the availability of the immediate riboflavin precursor GTP synthesized from amino acids, tetrahydrofolate derivatives, and CO_2_ via serine, threonine, and glyoxalate cycles is a major rate-limiting factor for riboflavin overproduction. Practically all upregulated reactions during the trophic phase of *A. gossypii* were involved in extracellular uptake of amino acids and nucleotides/nucleosides, including those of partially broken mycelia after autolysis (Ledesma-Amaro et al., 2014). However, an excess of extracellular purines represses the transcription of genes required for ATP and GTP synthesis by feedback inhibition of the *de novo* purine pathway. Similarly, excess serine and threonine have the same influence. Therefore, riboflavin overproduction in *A. gossypii* achieved via deregulation of the purine pathway at different levels to increase the glycine pool for GTP. Transcriptionally downregulated reactions were mostly used in relation to biomass formation, prevention of riboflavin consumption, and glycine degradation (Lim et al., 2001; Schlüpen et al., 2003; Jiménez et al., 2005; Park et al., 2011; Ledesma-Amaro et al., 2014; Revuelta et al., 2016).

Regulation of metabolic pathways by supplementation of structural analogs of metabolites (antimetabolites) inhibiting metabolic reactions is used to search for limiting steps of biosynthesis and ways to overcome them, including development of strain antimetabolite resistance (Schmidt et al., 1996; Park et al., 2007; Tajima et al., 2009). Antimetabolites, such as tubercidin blocking purine biosynthesis in *C. famata*, and itaconate and oxalate inhibiting isocitrate lyase for fatty acid use in *Aphis gossipii* are employed for selection of fungal riboflavin overproducing strains (Park et al., 2007; Tajima et al., 2009; Stahmann et al., 2000; Aguiar et al., 2015). Thus, oxalate resistance downregulated the expression of aldose reductase and methionine synthase that allows the strain to intracellularly accumulate glycine. Overexpression of malate synthase from the natural oxalate-resistant *A. gossypii* strain AgOXA50, using an oxalate-containing medium, improved riboflavin productivity fivefold (Sugimoto et al., 2010; Table 1). However, this was the first study that described the natural isolation of riboflavin overproducer (Sugimoto et al., 2010; Table 1). A mutant strain, which was yellow on itaconate-containing agar, produced 15% more enzyme and 25-fold more riboflavin (Sanchez and Demain, 2008; Table 1). The mutation of *A. gossypii* causing resistance to a glycine antimetabolite, aminomethylphosphonic acid, yielded improved producers (Sugimoto et al., 2010).

For *B. subtilis*, mutants resistant to purine analogs 8-azaguanine, methionine sulfoxide, and decoyinine increased GTP and riboflavin production because of deregulation in the purine pathway (Schwechheimer et al., 2016). Exposure to the riboflavin analog roseoflavin isolated from *Streptomyces davawensis* was found to lead *B. subtilis* to spontaneous mutations and constitutive riboflavin overproduction (Ludwig et al., 2018). Roseoflavin negatively affects FMN-specific *rib* operon regulators (FMN riboswitches) and flavoenzymes in bacteria, and is used together with multiple copies of *rib* operon genes to select their overproducing strains (Acevedo-Rocha et al., 2019).

**FIGURE 2:**
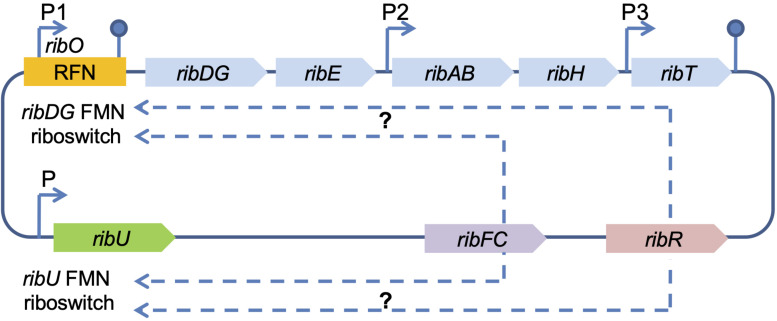
Scheme of the riboflavin biosynthesis regulation in *B. subtilis*. RFN, chromosomal FMN-specific element; FMN riboswitch, coding sequence for FMN binding; *ribDG-E-AB-H-T*, *rib* operon; *ribU*, gene encoding riboflavin transporter; *ribU* FMN riboswitch, the second chromosomal FMN-specific element (RFN); *ribFC*, gene encoding bifunctional flavokinase/FAD synthetase RibFC; *ribR*, gene encoding monofunctional flavokinase RibR (the part of a transcription unit encoding proteins for sulfur uptake and degradation), possibly involved in the regulation of the riboflavin biosynthesis genes (indicated by dashed arrows); P1, P2, and P3 denote confirmed promoters; P – predicted promoter (indicated by arrows). The hairpins symbols denote confirmed transcription terminators.

In contrast to fungi, riboflavin synthesis regulation in *B. subtilis* occurs by feedback repression of the *rib* operon via the riboswitch FMN-specific element (RFN) (Jiménez et al., 2005; Ledesma-Amaro et al., 2015; Pedrolli et al., 2015). It is a highly conserved RNA motif selective for the cofactor FMN, which modulates the expression of the FMN synthesis-associated genes (mostly transporters) in response to elevated concentrations of corresponding cellular metabolites (Meyer et al., 2015; Pedrolli et al., 2015). The *B. subtilis rib* operon consists of five genes, *ribDG, ribE, ribAB, ribH, ribT*, forming one transcription unit (mRNA), and contains the regulatory region *ribO*, untranslated leader region, that is located with the major promoter P1 (transcription start) upstream of the first structural gene in the operon (Figure 2) (Sklyarova et al., 2012). *RibO* together with P1, includes the so-called RFN element (FMN riboswitch/*ribDG* FMN riboswitch), which is involved in FMN biosynthesis. The *ribDG* FMN riboswitch regulates the expression of this gene cluster by binding FMN at a high cytoplasmic level (Pedrolli et al., 2015). Two additional internal promoters P2 and P3 of the *rib* operon are located in the regions of *ribE*, *ribH*, and *ribT* genes (Figure 2). Another RFN region (*ribU* FMN riboswitch) is located upstream of the 5′-end of the *rib* operon. The *ribU* gene encodes a transmembrane transporter for exogenous riboflavin uptake and flavin metabolism (Figure 2) (Rodionova et al., 2017). Thus, proteins for transport and biosynthesis are synthesized in parallel to ensure availability of the vitamin (Hemberger et al., 2011; Pedrolli et al., 2015). RibU-mediated riboflavin uptake was sensitive to protonophores and reduced in the absence of glucose, demonstrating that the protein requires metabolic energy for substrate translocation (García-Angulo, 2017).

In addition, the regulatory function in *B. subtilis* relates to *ribFC* and *ribR* genes (Figures 1, 2). The gene *ribFC* of bifunctional flavokinaze/FAD-synthase is not linked to the riboflavin operon and does not interact with the *ribO* region, but it is located elsewhere in the chromosome (Pedrolli et al., 2015). Subsequently, mutations of *ribFC* led to an increase of riboflavin concentration up to 15 g/L (Karelov et al., 2011). *RibR*, an RNA-binding protein that is also not part of the *rib* operon, is believed to act as a regulatory protein as it seems to interfere with the FMN riboswitch function (Higashitsuji et al., 2007; Pedrolli et al., 2015). The gene *ribR* encodes a monofunctional flavokinase as a part of the transcription unit consisting of 12 genes, whose products are involved in sulfur uptake and degradation. The *ribR* induction and repression occurred under methionine or taurine, and MgSO_4_, respectively (Pedrolli et al., 2015). Recently, it has been shown that when sulfur is present, *ribR* expression increases to block FMN riboswitches, the FMN demand of the cell increases, and the *rib* operon is expressed even with high FMN levels (Higashitsuji et al., 2007; Pedrolli et al., 2015). In *E. coli*, the bifunctional riboflavin kinase/FMN adenylyltransferase is encoded by *ribF*, which is analogous of *B. subtilis ribFC* and an essential gene for growth and survival (Figures 1, 2). By modulating *ribF* expression through mutations in its ribosome binding site and optimizing fermentation conditions, riboflavin production was improved by 12-fold up to a yield of 2702.8 mg/L in *E. coli* (Lin et al., 2014; Table 1). In a study conducted by Wang et al. (2015), the His115Leu mutation in bifunctional riboflavin kinase/FMN adenylyltransferase reduced enzyme activity to 55%, which was a probable reason for riboflavin accumulation in *E. coli* BL21. However, the exact regulation mechanism of riboflavin in *E. coli* is yet to be revealed. The *ribT* function remained unknown until recent research showed that its enzyme is a member of GCN5-related N-acetyltransferase, which transfers the acetyl group from acetyl-CoA to a variety of substrates (Srivastava et al., 2018; Figure 2).

The *rib* operon has also been studied in *Bacillus amyloliquefaciens*, *Bacillus halodurans, Bacillus abortus, Vibrio vulnificus, Shewanella oneidensis, Actinobacillus pleuropneumoniae*, *C. glutamicum*, and *Bartonella* spp. (García-Angulo, 2017; Vitreschak et al., 2002). In *Photobacterium phosphoreum* and *Photobacterium leiognathi*, riboflavin genes are localized within the *lux* operon (Vitreschak et al., 2002). In contrast, *E. coli* genes are not clustered in an operon, but are scattered on the chromosome (Palacios et al., 2014; García-Angulo, 2017). Moreover, bacterial *rib* operons may also include genes other than *rib* (García-Angulo, 2017). The regulatory RFN-elements are found on the chromosome of numerous, but not all, bacterial species. Interestingly, no clear phylogenetic distribution was found for these genes. Species can either have both transporter and biosynthesis genes (*L. plantarum, Pediococcus pentosaceus, B. subtilis, Staphylococcus aureus*), only one of the two (*Lactobacillus johnsonii, Lactobacillus brevis, Lactobacillus delbrueckii, Streptococcus pneumoniae, Enterococcus faecalis*), or lack both systems (*Listeria monocytogenes, Lactobacillus casei*). Notably, the RFN element was not found in front of all transport units encoding the presumed riboflavin transporter (Wels et al., 2006). Only spirochetes, mycoplasmas, and rickettsia have neither riboflavin genes nor RFN elements (Vitreschak et al., 2002; Burgess et al., 2004).

## Riboflavin-Producing Strain Improvement

Attempts to improve microbial riboflavin-producing strains were made by both metabolic and genetic engineering, which include the following: (1) random mutagenesis by chemical exposure and UV irradiation (Matsuyama et al., 1987; Park et al., 2007; Kavitha and Chandra, 2014); (2) random and site-directed mutagenesis by genetically engineered deletions, insertions, or substitutions (Monschau et al., 1998; Zamboni et al., 2003; Jiménez et al., 2005, 2008; Zhu et al., 2006; Kato and Park, 2012; Lin et al., 2014; Ledesma-Amaro et al., 2015; Wang et al., 2015; Liu et al., 2019; Lu et al., 2019); (3) selection (Schmidt et al., 1996); and (4) optimization of the fermentation process depending on medium components and their concentrations (Leathers and Gupta, 1997; Kalingan and Liao, 2002; Lim et al., 2003; Pessoa et al., 2003; Wu et al., 2007; Sugimoto et al., 2010; Cheng et al., 2011; Suzuki et al., 2011). Duplications, insertions, deletions, modifications, substitutions, upregulations, and downregulations of genes directly or indirectly associated with riboflavin biosynthesis were often combined by manipulation with nutritional and other growth factors (Ledesma-Amaro et al., 2014, 2015; Revuelta et al., 2016; Schwechheimer et al., 2016). Numerous physiological and genetic methods have been developed to enhance production of defined secondary metabolites, allowing for an increase in riboflavin yield. Mutations of key genes and non-coding regions in microbial genomes has facilitated overproducing strain development (Park et al., 2011; Li et al., 2013; Ledesma-Amaro et al., 2014, 2015; Liu et al., 2019; Lu et al., 2019).

Improvement of the producer most often begins with random mutagenesis and routine screening for mutants by qualitative and quantitative determination of riboflavin (Table 2). Screening of mutants may include determining the productivity of up to several thousand colonies after each round of mutagenesis (Park et al., 2011; Lin et al., 2014; Ledesma-Amaro et al., 2015). This approach is particularly useful when there are no data on which specific gene or region of the genome would result in the desired phenotype upon mutation. The random ninefold upregulation of genes involved in purine and riboflavin pathways was reached after the use of lagging-strand-biased mutagenesis (disparity mutagenesis) toward *A. gossypii* (Table 2; Park et al., 2011).

**TABLE 2:** Genetic modification methods used for the riboflavin-producing strains.

Random mutagenesis	Site-directed mutagenesis
Disparity mutagenesis (the lagging-strand-biased-mutagenesis) – a mutation is inserted into DNA polymerase δ which is responsible for synthesis of the lagging strands, losing its DNA repair function (Park et al., 2011). Mutagenesis by ultraviolet (UV) radiation (Park et al., 2007; Tajima et al., 2009; Silva et al., 2019) Mutagenesis by chemical mutagen, N-methyl-N′-nitro-N-nitrosoguanidine (NTG) (Matsuyama et al., 1987; Tajima et al., 2009). Mutagenesis by hydrogen peroxide and menadione (Walther and Wendland, 2012; Kavitha and Chandra, 2014).	Vectors constructions and their transformation into cells (bacteria), protoplasts or conidia (fungi) (Santos et al., 1995; Debabov et al., 1997; Monschau et al., 1998; Perkins et al., 1999a,b; Koizumi et al., 2000; Babyak et al., 2002; Zamboni et al., 2003; Lee et al., 2004a,b; Althofer et al., 2005; Jiménez et al., 2005, 2008; Mateos et al., 2006; Zhu et al., 2006; Marx et al., 2008; Duan et al., 2010; Kato and Park, 2012; Ledesma-Amaro et al., 2014, 2015; Lin et al., 2014; Park et al., 2014; Silva et al., 2015; Wang et al., 2015; Srivastava et al., 2018; Liu et al., 2019; Lu et al., 2019). *CRISPR*/*Cas9* genome editing *technology* (Liu et al., 2019; Lu et al., 2019).

However, random mutagenesis may not reveal a mechanism for increasing strain productivity that is additionally unstable in contrast to site-directed mutagenesis, which implies the presence of a target nucleotide sequence with a known function. By site-directed mutagenesis, it is possible to obtain stable and reproducible mutants with predictable gene expression regulation related to riboflavin biosynthesis (Table 3). Site-directed mutagenesis is often applied to a strain obtained by random mutagenesis to optimize growth and create an overproducer. Bacterial and fungal riboflavin biosynthetic pathways, as well as molecular-genetic strategies and toolboxes for riboflavin-producing capability improvement are different (Figure 1 and Table 3). Riboflavin synthetic genes have been studied more extensively in *E. coli* and *B. subtilis* (Revuelta et al., 2016; Schwechheimer et al., 2016).

**TABLE 3:** Strategies and genetic tools for the riboflavin-producing strains improvement.

Wild-type strain (mutant strains)	Strategies and tactics	Genetic tools and methods	Results and conclusions	References
*A. gossypii* ATCC 10895 (*A. gossypii* pAG203GLY1).	Activation of the purine pathway by: (1) Overexpression of the threonine aldolase *GLY1* gene for the formation of glycine from threonine as an early precursor required for purine synthesis; growth on 1% yeast extract, 1% glucose supplemented with 50 mM threonine.	The *A. gossypii* gene *gly1* (1146 bp ORF) was inserted into the expression plasmid pAG203 by the added sites *Sph*I-*Sca*I under the control of the constitutive TEF promoter and terminator; Mycelium electroporation, geneticin-resistant spores selection (1.8 mg/ml geneticin).	(1) 10-fold increase in threonine aldolase specific activity; (2) Increase of glycine concentration from 2 ± 0.2 to 41 ± 4 mM; (3) Eightfold increase in riboflavin production: ≥17 ± 3 mg per mycelial dry weight (mdw) after 3 days of cultivation.	Monschau et al., 1998
*A. gossypii* ATCC 10895 (the overexpressing strain *GPD-ADE4*-; the mutant strains *GPD-ade4-VQ* and *GPD-ade4-WVQ*).	Activation of the purine pathway by: (1) Overexpression of the PRPP amidotransferase *ADE4* gene for àbolishing the metabolic regulation of the committed step catalyzed by the enzyme PRPP amidotransferase. (2) Designing the mutant enzyme resistant to feedback inhibition by purine derivative monophosphates (AMP, GMP, ATP, and GTP) or the deletion of the enzyme gene.	(1) For gene disruption: Ag*ADE4* ORF replaced by G418^*r*^ cassette with flanked by PCR-based 50 bp of 5′- and 3′-flanking regions. (2) The overexpression module, including the *A. gossypii* gene Ag*ADE4* (1533 bp, *accession no.* A94856) under the control of a strong 400-bp constitutive promoter of glyceraldehyde-3-phosphate dehydrogenase (Ag*GPD)* placed 9 bp before the ATG of Ag*ADE4* gene, was cloned into the added *Nco*I site of plasmid pGEM-T. The overexpression modules (including mutant *GPD-ade4-VQ*, and *GPD-ade4-WVQ with* K^333^/Q^333^ and D^310^/V^310^ substitutions) were transformed into AgΔade4 spores. Growth on a medium supplemented with 100 mg/L adenine and G418 as selective markers.	(1) 2.7-fold (77.2 mg/L) and 10-fold (228 mg/L) riboflavin production enhancement in the *GPD-ADE4*-and *GPD-ade4-WVQ* strains, respectively (the wild-type *A. gossypii* ATCC 10895 riboflavin production – 28 mg/L).	Jiménez et al., 2005
*A. gossypii* ATCC 10895 (the overexpressing strains *GPD-PRS2,4* and *GPD-PRS3; the mutant strains prs2,4-IQ and prs3-IQ*).	Alterations in PRPP synthetase (PRS) activity, controlling the purine precursor PRPP, and involved in the *de novo* and *salvage* biosynthesis of GTP by: (1) *Disruption and overexpression* of two PRPP synthetases genes (*AGR371C* and *AGL080C*, in the AGD database http://agd.vital-it.ch/index.html). (2) Deregulation of the enzymes PRSs that inhibited by ADP with the use of PCR-based site-directed mutations Leu^133/132^/Ile^133/132^ and His^196/195^/Glu^196/195^ in accordance to the PRPP synthetase superactivity in humans.	*For gene disruption:* (1) A *kanMX4* module with *G418*^*r*^ marker of the plasmid pAG-110 (by *Sal*I ends) blunt-ended and inserted between *Hin*cII and *Eco*RV sites in the *AGR371C* ORF, and digested with *Nco*I and *Kpn*I for the spores transformation. (2) A *Hyg*^*r*^ resistance marker obtained with *Bam*HI-*Kpn*I ends blunt-ended and inserted between two *Eco*RV sites in the *AGL080C* ORF, and digested by *Eco*RI for the spores transformation. (3) For overexpression: the *AGR371C* and *AGL080C* ORFs inserted as an *Nde*I-*Bam*HI fragment into the cassette allowing stable genomic integration into the *AgLEU2* locus described by Jiménez et al. (2005). Growth on a medium supplemented with ADP and G418.	(1) Increased mRNA levels of both genes by 30-fold. (2) The riboflavin productivity of the overexpressed *AGR371C* (*GPD-PRS2,4*) and *AGL080C* (*GPD-PRS3*) strains – 42.4 mg/L and 40.4 mg/L, respectively, indicating a posttranslational regulatory mechanism of the enzymatic activity. (3) In the mutant strains – 80% greater the enzymatic activity in the presence of repressor ADP, however, the riboflavin production were the same as in the overexpressed PRSs strains.	Jiménez et al., 2008
*A. gossypii* ATCC 10895 (the mutant strains Δ*bas1* and ΔC631BAS1).	Constitutive activation of the purine and glycine pathways for the high GTP levels by: (1) Deletion C-terminal regulatory domain of BAS1 sensitive to the high concentration of GTP (630 to 664 aa according to BIRD domain of *S. cerevisiae* BAS1) in Ag*BAS1* (ID: *AFR297W* in http://agd.unibas.ch/), encoding the Myb family transcription factor involved in the regulation of purine and glycine biosynthesis, riboflavin overproduction, and growth.	(1) For insertional mutagenesis: *A. gossypii* genomic DNA digested with *Pst*I and a minitransposon *R* comprising the 5′ and 3′ terminal repeats from the *Himar1* transposon flanking the G418r marker and the bacterial replicon ColE1. Transform the *E. coli* DH10B by the self-ligated genomic DNA with the integrated minitransposon R to obtain the plasmid library that linearized by *Pst*I digestion to transform *A. gossypii*. (2) For disruption (construction of Δ*bas1*): The *Bam*HI-*Sph*I fragment of the Ag*BAS1* ORF replaced by *G418*^*r*^ marker, and *Xho*I-*Bgl*II digested with a 356-bp 5′- and a 520-bp 3′-flanking regions homologous to the Ag*BAS1* locus to transform spores. For expression of the truncated Ag*BAS1* (1-305 aa DNA-binding domain); construction of Δ*C631BAS1*: A PCR-derived module containing the 50-bp fragment upstream from the 631 aa codon of AgBas1 followed by the *ScADH1* terminator, the *G418*r marker, and the 50-bp fragment downstream from the Ag*BAS1* stop codon to transform *A. gossypii* spores and to integrate in the *BAS1* locus.	(1) Bas1-independent basal transcription of the *de novo* purine genes in Δ*bas1* strain, but only in the presence of extracellular adenine. (2) The truncated ΔC631Bas1 form is insensitive to the high GTP levels and induces a constitutive transcriptional activation of *ADE4* and *SHM2* insensitively from extracellular adenine. (3) The riboflavin production of the wild-type *A. gossypii* – 2.58 ± 0.13 mg/g of biomass; In Δ*bas1* strain: 15.31 ± 0.23 mg/g of biomass; In ΔC631BAS1 strain: 12-fold increased in riboflavin production – 24.28 ± 0.37 mg/g of biomass after the 96-h cultivation.	Mateos et al., 2006
*A. gossypii* ATCC 10895 (mutant strains AgΔSHM1 and AgΔSHM2).	Activation of the glycine pathway by: (1) Disruption of the SHM1 and SHM2 genes (the EMBL Data Bank accession n. AJ438778 and AJ438779) encoding two isozymes of serine hydroxymethyltransferase for reducing carbon flux from glycine to serine.	Ashbya genomic library constructed in the cosmid vector SuperCos1 (Stratagene) screened for the positively probed enzyme-containing fragments (pJR clones). For SHM1 disruption, a 769-bp *Xho*I ± *Sal*I part of AgSHM1 ORF was replaced with a 2.1-kb G418^*r*^ cassette. The 2.7-kb *Bam*HI ± *Kpn*I digested plasmid pJR1550 SHM1769: G418 was used to transform *A. gossypii*, inducing DNA integration by homologous recombination. (1) For SHM2 disruption: a 1.3-kb *Sal*I ± *Eco*RV part of the plasmid pJR2417 was deleted, and a 1.6-kb *Bam*HI ± *Hin*dIII fragment containing the Hyg^*r*^ marker was inserted. A 2.1 kb linear fragment containing the SHM2D1300: Hyg^*r*^ marker was obtained by the *Sph*I digestion of the plasmid pJR2427 to transform *A. gossypii*.	(1) AgDSHM1 produced the same amount of riboflavin (1.1 ± 0.2 mg/g biomass) as the wild-type (0.9 ± 0.1 mg/g biomass), the production of AgDSHM2 increased 10-fold (9.6 ± 1.0 mg/g biomass). (2)^13^C-labeling experiments proved the shift metabolic pathway from serine to glycine biosynthesis in the mutant strain AgΔSHM2.	Schlüpen et al., 2003
*A. gossypii* ATCC 10895 (the multiple-engineered *Ashbya* strain A330).	Activation of the RIB genes and the AMP branch of the purine nucleotide biosynthetic pathway by: (1) Overexpression of the RIB genes. (2) The inactivation and the underexpression of the ADE12 gene, which controls the first step of the AMP branch.	(1) For gene overexpression: the AgGPD promoter integrated upstream of the ATG initiator codon of each gene. An overexpression cassette comprising the AgGPD promoter (P) and the loxP-KanMX-loxP selectable marker (G418r), was PCR-amplified using specific primers for each gene. The loxP repeated inverted sequences enabled the selection marker to be eliminated, and subsequently reused, by expressing a Cre recombinase after each round of transformation. The quintuple RIB-engineered strain, which overexpresses the RIB1, RIB2, RIB3, RIB5, and RIB7 genes was obtained after 10 transformations either to integrate the AgGPD promoter into the target loci or to remove the KanMX selection marker. (2) For the deletion of AgADE12 (ade12Δ): a gene replacement cassette was constructed by PCR amplification of the loxP-KanMX-loxP marker flanked by ADE12-flanking recombinogenic sequences to transform *A. gossypii*. The homokaryon clones were isolated by sporulation of the primary transformants. (3) For *ADE12* gene underexpression: the native promoter was replaced by the weaker (by 62-fold) promoter of the RIB7 gene using a *loxP*-*KanMX*-*loxP*.	(1) The ade12Δ strain produced 246 mg/L (2.5-fold increased compared to the wild-type strain), but showed adenine auxotrophy. (2) The mRNA levels of ADE12 were reduced 70-fold in the P_*RIB*__7_-ADE12 strain, but sufficient without adenine supplementation and similar in the riboflavin yield with ade12Δ. (3) The strain A330 modified both for the underexpression of the ADE12 gene (P_*RIB*__7_-ADE12) and for the overexpression of five RIB genes afforded the highest riboflavin yield. This strain produced 523 mg/L of riboflavin (5.4-fold higher than the wild-type).	Ledesma-Amaro et al., 2015
*A. gossypii* ATCC 10895 (ΔIMPDH and P GPD – IMPDH strains).	Activation of the metabolic flux through the guanine nucleotide pathway (the rate-limiting step) by: (1) The overexpression of the IMP dehydrogenase (AgIMPDH) that catalyzes the reaction at the branch point between the guanine and adenine nucleotide biosynthetic pathways.	(1) For AgIMPDH gene disruption (Δ*IMPDH* strain): replacement DNA cassette containing the *kanMX4* selection module including G418r and flanked by specific homology regions was transformed into the spores. (2) For AgIMPDH gene overexpression (*P GPD –IMPDH strain)*: the AgIMPDH ORF inserted into a DNA cassette comprising a module for G418r, a recombination module for stable integration into the *STE12* locus (does not affect inosine, guanosine, riboflavin production, or growth rate), and an overexpression module based on the strong constitutive *A*. *gossypii* glycerol 3-phosphate dehydrogenase promoter (*P GPD*) and terminator.	(1) Δ*IMPDH* strain showed the 20-fold increase in the inosine production and decrease guanosine and riboflavin levels, and auxotrophy for guanine (growth using the action of the *salvage* pathway). (2) IMPDH disruption results in a 100-fold increase of inosine excretion to the culture media. (3) *IMPDH* overexpression significantly decreased inosine excretion, while the guanosine levels remained constant, and enhanced about 40% riboflavin production.	Buey et al., 2015
*A. gossypii* ATCC 10895 (the mutant strain W122032).	Activation of purine and riboflavin biosynthetic pathways by: the mutation of POL3 gene, encoding DNA polymerase δ responsible for the constitutive DNA reparation, might positive modulate carbon flow toward the purine and riboflavin synthetic pathways.	Genetic mutation technology – disparity mutagenesis. (1) The mutant-inducing vector YCpG418/poldexo construction: LEU2 (1.2 kb) of YCplac111 (Gietz and Sugino, 1998) was *Aat*II and *Eco*RV excised, G480r cassette (2.5 kb) inserted into the *Bam*HI site of MCS in YCpG418 plasmid. (2) The POL3 gene (AFL189W) including 3.3 kb ORF, 1 kb promoter and 0.6 kb terminator were mutated using PCR: 946 bp (A→C) and 952 bp (A→C). The resulting mutated POL3 (4.9 kb) inserted into the *Xba*I site of YCpG418 (the YCpG418 / poldexo- plasmid) was transformed into *A. gossypii* by electroporation. (3) Until the 18th generation, YR medium was used; from the 19th to 30th generation, YR containing 2% rapeseed oil and 3% yeast extract was used to avoid nutrient depletion. The test tube cultures were carried out at 28°C with 150 rpm for 24 h.	(1) Among 1353 colonies generated in the first screen, 26 mutants produced more than 3 g/L of riboflavin. (2) By the second screen and single-colony isolation, nine strains produced more than 5.2 g/L of riboflavin. The strains were resistant to oxalic acid and hydrogen peroxide as antimetabolites. (3) The final strain W122032 produced in a 3-L fermentor 13.7 g/l of riboflavin in an optimized medium. (4) Expression of the purine and RIB genes, particularly *ade*1, *rib*1, and *rib*5, more than twofold higher, RIB1 and RIB3 were expressed with sixfold higher levels. (5) While carbon source assimilation, energy generation, and glycolysis were downregulated at the riboflavin-producing phase.	Park et al., 2011
*B. subtilis* strain 3979 (the high-performance riboflavin production strain BSHP (*B. subtilis* < pHT01ribM_*opt*_ >).	Reduction of the riboflavin levels in the cytoplasm enhancing the carbon flux through the riboflavin biosynthesis pathway by: Introducing the transport system for flavins that oxidatively damage the bacillus cells, thus limiting their intracellular synthesis.	*B. subtilis* strains overexpressing the codon-optimized ribM_*opt*_ gene (GenBank FR719838) were generated using expression vector pHT01 (Mobitech, Göttingen, Germany) based on the bacillus pUB110 plasmid and used as *E. coli* – *B. subtilis* shuttle vector. 0.01–1.0 mM IPTG, 30 μg ml^–1^ chloramphenicol, and 10–100 μM roseoflavin as selective antimetabolite were added to the growth medium for 30-h cultivation.	(1) Transport protein RibM from *S. davawensis* mediates flavin (riboflavin/roseoflavin) translocation *via* an energy independent facilitated diffusion mechanism. (2) The strain BSHP produced about 350 mg/L riboflavin. (3) The overproduction of RibM allowed growth of a ΔribU:Kanr ΔribB:Ermr *Bacillus subtilis* strain.	Hemberger et al., 2011
The recombinant strain *Bacillus subtilis* RH33 [the recombinant strain *B. subtilis* PY with modified riboflavin operon; *B. subtilis* PYZ with an additional structural gene (*zwf*) gene in *zwf* locus].	The modulation of pentose phosphate (PP) pathway by: (1) Overexpression of glucose-6-phosphate dehydrogenase (G6PDH). (2) By the further improvement of riboflavin producer *B. subtilis* RH13 containing the integrative plasmid pRB63 and autonomous plasmids pRB49, pRB62 with bacillus riboflavin operon and producing 0.4 g/L of riboflavin.	(1) The modification of the heterologous riboflavin operon of *Bacillus cereus* ATCC14579 was carried out by replacing its native promoter with a strong constitutive promoter P43 to randomly insert in the chromosome (strain PY). (2) For overexpression of G6PDH, the integration plasmid having both Pxyl inducible promoter and the coding sequence of the structural gene *zwf* from *B. subtilis* 168 (http://www.ncbi.nlm.nih.gov/), cloned into the *Bam*HI-*Sma*I site of pUC18 together with spectinomycin resistance cassette from pSG1192 (BGSC, Bacillus Genetic Stock Center). The plasmid were integrated into the *zwf* locus of *B. subtilis* chromosome by crossover homologous recombination (the strain PYZ).	(1) The PP pathway fluxes are increased in response to overexpression of G6PDH that associated with an increased intracellular pool of Ribu5P, a precursor for riboflavin biosynthesis. (2) Overexpression of G6PDH resulted in the glucose consumption rate increasing slightly, while the specific growth rate was unchanged. (3) An improvement by 25% ±2 of the riboflavin production in the strain PYZ and 0.04 g per gram in the strain PY.	Duan et al., 2010
*B. subtilis* 168 (the mutant strain *B. subtilis* PK).	The carbon flux redistribution with higher flux to PP pathway: by disruption of the low coupling *bd* oxidase.	Expression of cytochrome *bd* requires *cydA* and *cydB*, which code for the two subunits of the enzyme as well as two additional genes, *cydC* and *cydD* (Winstedt et al., 1998). To construct a *cydABC* deletion–insertion mutation, the primers cydA+(CCCGGGTCGGTGTTGTAAC) and cydC−(CCCGGGGGATCCTCCCGCTGAGGCAG) were designed using the sequence of the *B. subtilis cyd* gene obtained from GenBank and used to amplify a 3.55-kb fragment from the genomic DNA of *B. subtilis* 168. The fragment was digested with *Eco*RI and *Bam*HI and cloned into *Hin*dIII-site-disrupted pUC18. After isolation and characterization of pUC-*cyd* plasmid, a chloramphenicol resistance gene (Cm^*r*^) was inserted in the middle of the cloned *cyd* DNA. The 1.2-kb chloramphenicol resistance cassette from plasmid pC194 was amplified using primers Cm^*r*^+(CCCGGGAAGCTTCGCTACGCTCAAATCCCTTTA) and Cm^*r*^−(CCCGGGAAGCCGACCATTC). After purification and digestion with *Hin*dIII, it was cloned into *Hin*dIII-digested pUC-*cyd* and gave plasmid pUCL37. *Sca*I-linearized pUCL37 was transformed into *B. subtilis* PK; transformants were selected on plates containing 5 μg of chloramphenicol/ml and then correct insertions were verified by PCR analysis.	About 30% higher precursor was availability for riboflavin biosynthes.	Li et al., 2006

The parent *B. subtilis* strain 168 and its siblings (strains 23, 122, 160, 166) for several riboflavin overproducers (Tables 2, 3) are those that have survived the earliest years of *B. subtilis* genetics following sub lethal doses of UV or X-rays caused by high frequencies of auxotrophy (a specific nutrient requirement for growth) and single nucleotide polymorphisms (SNPs) (Zeigler et al., 2008). Although they initially originated from the parent variant selected for the fastest growth on glucose-ammonia minimal medium, auxotrophs required threonine (strain 23), nicotinic acid (strain 122), or tryptophan (strains 160, 166, and 168) due to damage of some key metabolic genes. For further optimization of *B. subtilis* industrial producers, pUC-based plasmids, and chromosomal homology recombination of these strains were employed (Table 3).

However, the industrial process of riboflavin biosynthesis in *B. subtilis* is still dependent on several barely resolved issues, including *RibR*-regulation of FMN riboswitches limiting its production; unknown riboflavin pathway phosphatases; flavin reactivity damaging cells; and the absence of a transport system to export actively flavins in contrast to that of *A. gossypii* (Acevedo-Rocha et al., 2019). Wild-type *B. subtilis* cells rapidly convert intracellular riboflavin to FMN and FAD catalyzed by the bifunctional flavokinase/FAD synthetase RibC and cannot actively export flavins like *A. gossypii*. Consequently, the introduction of the gene *ribM* from *S. davawensis*, encoding the energy independent flavin transport-catalyzing protein, enhanced roseoflavin sensitivity and riboflavin export from their cytoplasm and increased riboflavin yield (Table 3; Hemberger et al., 2011).

However, most effort was applied to regulation modification of the *B. subtilis rib* operon and overexpression of structural genes *ribDGEABH* (Table 3; Abbas and Sibirny, 2011; Lee, 2015). Highly effective riboflavin production strains were constructed by introducing additional copies of *ribDGEABH* genes controlled by strong native or strength-evolved synthetic bacterial and phage promoters (Lee, 2015; Cisternas et al., 2018; Han et al., 2019).

The first riboflavin operon, encoding riboflavin biosynthesis genes, and overproduction in *B. subtilis* were studied at the Russian Institute of Genetics and Selection of Industrial Microorganisms. The genetically engineered *B. subtilis* strain VNIIGenetika 304/pMX45 produced 4.5 g/L of riboflavin after 25 h of fermentation, but was not stable due to the presence of repeated chromosomal and episomal copies of the *rib* operon (Lee, 2015). Further works on *rib* operon replacement from *B. amyloliquefaciens* led to the successful development of the *B. subtilis* strain GM41/pMX4557, which accumulated up to 9 g/L riboflavin. Sequencing the *B. subtilis rib* operon gave rise to new approaches for construction of novel riboflavin-producing strains (Hohmann et al., 2010). Gene amplification and replacement of wild-type promoters and regulatory regions with a strong constitutive promoter from the *Bacillus* bacteriophage *SPO1* have resulted in a strain with remarkably improved riboflavin production up to 15 g/L. Perkins et al. (1999a, b) claimed the process for riboflavin production using the *B. subtilis* strain, requiring at least, a deregulation of the purine synthesis and a mutation in flavokinase/FAD-synthase (Shi et al., 2014; Table 1). As a result, recombinant *B. subtilis* has been shown to be usable in large-scale fermentations and riboflavin production in amounts greater than 15 g/L (Perkins et al., 1999a,b; Lee et al., 2004a,b; Lee, 2015; Wu et al., 2007). The well-known *B. subtilis* mutant RB50:(pRF69)6o(Ade+), containing a transcriptionally-modified riboflavin operon with two SPO1-15 promoters, produced 13.0–14.0 g/L riboflavin in 48 h and 15 g/L in 56 h during cultivation in standard commercial batch and feed conditions (Table 1).

The genetically improved *B. subtilis* riboflavin overproducing strains seem to be used for fermentation products placed on the EU market as a feed additive. Recently, the multiplied genetically modified strain has been identified and isolated from the vitamin B2 product (80% feed grade) imported from China due to the development of whole genome sequencing (WGS) (Paracchini et al., 2017). The WGS data revealed the integration of a marker resistance gene, the deletion of the endogenous *rib* operon, and the presence of four putative recombinant pBR322 and pUC-based plasmids harboring additional *rib* operons. Four chromosomal deletions involved an integrative and conjugative element (ICE) in the *trnS-leu2* gene; the *ribD*, *ribE*, and *ribAB* genes in combination with the flavin riboswitch. A crossing over recombination insertion in the chromosome contained the chloramphenicol resistance gene *cat* (ENA ID: LT622644) and disrupted the gene *recA* (*recE*), encoding a multifunctional protein for homologous recombination and DNA repair. Finally, when compared to the *B. subtilis* strain 168, more than 400 potential SNPs were identified. The plasmid pGMBsub01 (ENA ID: LT622641) contained the full *B. amyloliquefaciens ribDGEABHT* operon, together with upstream and downstream genes of segregation proteins (ScpA, ScpB) directly linked to the *S. aureus* replication initiation protein B (RepB). The pGMBsub02 plasmid (ENA ID: LT622641) carried only *ribD* and *ribE* genes from the *B. amyloliquefaciens rib* operon. The pGMBsub03 plasmid (ENA ID: LT622642) included part of the *B. subtilis rib* operon (*ribA, ribH, ribT*). The pGMBsub04 plasmid (ENA ID: LT622643) included the full *B. subtilis ribDGEABHT* operon together with the genes from *Enterococci* and *Streptococci* plasmids, where the non-*rib* operon sequence was identical to the plasmid pSM19035 (GenBank ID: AY357120), a low-copy-number theta-replicating plasmid of *Streptococcus pyogenes*, stably maintained in a broad range of Gram-positive bacteria. All of the plasmid sequences were characterized by the presence of several selective antibiotic resistance genes from pUC19, pUB110, and pSM19035 vectors. The deletion of the endogenous *ribDGEABHT* operon indicates that the strain is unable to produce riboflavin without recombinant plasmids encoding the *rib* operon. Paracchini et al. (2017) have developed event- and construct-specific real-time PCR methods for detection of the GM strain and its putative plasmids for food and feed products.

The molecular toolbox for site-directed mutagenesis available for *A. gossypii* modification is still limited due to the lack of knowledge regarding fungal genomics and metabolomics. The Cre-loxP recombination system of bacteriophage P1 is commonly used to mediate recombination between repeated loxP sites flanking selectable markers in *S. cerevisiae* homologous to *A. gossypii* (loxP–marker gene–loxP cassettes), which allows removing and reusing marker genes as PCR-based targeting tools (Table 3). The cassette containing amplified DNA for replacement is flanked by loxP sequences and guide sequences with homology to the 5′- and 3′-untranslated regions (UTRs) of target loci for correct location and direction in the genome. Thereafter, it is transformed into homokaryotic spores by a Cre (recombinase)-expressing plasmid to introduce the deletion or mutation into target genes for their functional study, as well as metabolic improvement (Aguiar et al., 2014; Table 3).

Target genes for modification are determined by metabolic engineering strategies based on the knowledge of riboflavin biosynthetic pathways in *A. gossypii* (Figure 1).

The overexpression of *gly1* in *A. gossypii*, encoding a threonine aldolase homologous to *S. cerevisiae*, provided the formation of an excess of glycine from the exogenous threonine following its supplementation to the growth medium to 50 mM, and improved riboflavin biosynthesis by eightfold (Table 3; Schwechheimer et al., 2016). Research conducted by Jiménez et al. (2005) and Silva et al. (2015) on metabolic engineering of the pentose phosphate, glycine, and purine pathways of *A. gossypii* describe the phosphoribosyl pyrophosphate (PRPP) synthetase and PRPP amidotransferase (ADE4) gene overexpression, which increased the carbon flux through the pentose phosphate and purine/GTP biosynthetic pathways (Table 3; Jiménez et al., 2008; Silva et al., 2015). Mateos et al. (2006) described the identification and characterization of the transcription factor Bas1 in *A. gossypii* that participates in regulated transcription of genes involved in the biosynthesis of purines and glycine (Table 3; Mateos et al., 2006). The C-terminal domain BIRD of Bas1 is sensitive to a high concentration of the direct riboflavin precursor GTP, where it binds to sites of ADE4 and serine hydroxymethyltransferase (SHM2) promoters to depress the *de novo* purine pathway. Therefore, BIRD domain inactivation or deletion could constitutively activate transcription of purine pathway genes and synthesize an excess of GTP, which must be detoxified via riboflavin overproduction. The different *bas1* mutants showed a significant increase in the production of riboflavin and other growth-related phenotypes (Table 3; Mateos et al., 2006). A successful strategy for increasing the glycine precursor supply was disruption of the SHM2 gene that codes for a serine hydroxymethyltransferase in *A. gossypii*, converting glycine into serine. SHM2-disrupted mutants had reduced activity of this transferase, thus leading to the metabolic shift from serine to the riboflavin precursor glycine and, consequently, to the 10-fold increase in riboflavin production (Table 3; Schlüpen et al., 2003). As low transcription activity of *RIB* genes, excluding *RIB4*, and competition of AMP branch for purinogenic precursors, two important rate-limiting steps of riboflavin production, the riboflavin titer in *A. gossypii* A330 was enhanced 5.4-fold by overexpression of all *RIB* genes and through enhancement of the GMP purine branch by reducing ADE12 gene expression, whose enzyme, adenylosuccinate synthase, controls the formation of AMP from IMP (Table 3; Ledesma-Amaro et al., 2015). Additionally, overexpression of the inosine-5′-monophosphate dehydrogenase (IMPDH) gene increased metabolic flux through the guanine pathway and ultimately enhanced riboflavin production by 40% compared to the wild-type *A. gossypii* (Table 3; Buey et al., 2015). The ninefold improvement in riboflavin production was observed in the *A. gossypii* strain W122032, modified by the mutated DNA polymerase δ, losing its DNA repair function and introducing errors in the lagging strand, and increasing the riboflavin yield up to 13.7 g/L for 9 days of cultivation in optimized medium containing waste edible oils. A shift in carbon flux from β-oxidation to the riboflavin biosynthetic pathway was proved by a twofold increase in *ADE1*, *RIB1*, and *RIB5* protein synthesis, and in gene expression of gluconeogenesis and pentose phosphate cycles, but it was observed that the downregulation of pathways were related to carbon source assimilation, energy generation, and glycolysis at the riboflavin-producing phase (Table 3; Park et al., 2011).

Currently, two modern effective CRISPR/Cas- and CRISPR/Cpf1-mediated genome-editing systems have been adapted for the industrial fungus *A*. *gossypii*, enabling the efficient introduction of deletions, insertions, and nucleotide substitutions (Jiménez et al., 2019a,b). The CRISPR/Cas9 strategy comprises expression modules for CAS9 nuclease and complex synthetic guide RNA (sgRNA). sgRNA expression is driven by regulatory sequences from the *A. gossypii* SNR52 gene, which is transcribed by RNA polymerase III. It can be challenging for the genomic addition of AT-rich regions. In contrast, the nuclease Cpf1 (recently renamed as Cas12a) from *Lachnospiraceae bacterium* displays additional ribonuclease activity that functions in CRISPR RNA (crRNA) processing, and is guided by a single crRNA (gRNA). Due to intrinsic Cpf1 ribonuclease activity that facilitates crRNA processing and an array of donor DNA sequences for homology-directed repair of double-strand breaks generated by Cpf1, the multi-CRISPR/Cpf1 system is more efficient for multiplex deletion of up to four genes (Jiménez et al., 2019a).

CRISPR-based genomic editing has been developed for multiplex gene editing in *Bacillus* (Liu et al., 2019; Lu et al., 2019). The improved CRISPR/Cas9n mediated multiplexing system reached an efficiency of 65% for three-point mutations in *rib*A, B, and H genes (Liu et al., 2019). Due to hierarchical gene regulation at multiple levels in a context-dependent manner, fine-tuning of gene expression is crucial for protein expression and pathway construction. The CRISPR-assisted simultaneous up- and downregulation of the different genes expression (promoter-based transcription, molecular chaperone-assisted protein folding, protease-mediated degradation) during expression of amylase BLA improve in *B. subtilis* to 260-fold yield value of the target product BLA in a single cycle (Lu et al., 2019).

## Industrial Production of Riboflavin and Its Applications

The technological process of fermentative riboflavin production is composed of three main steps: upstream processing, bioprocess or fermentation, and downstream processing. Upstream processing includes strain development, sterilization of carbon and nitrogen sources, medium and inoculum preparation. The next step is actual fermentation processing. It runs under optimal pH, temperature, aeration, and agitation rates. Downstream processing includes broth pasteurization, isolation, purification, recrystallization, and drying of riboflavin (Heinzle et al., 2006).

The annual total riboflavin market is approximately 9000 t, and the final price is approximately $15/kg for the feed-grade product and $35–50/kg for the food-grade product (Abbas and Sibirny, 2011; Kato and Park, 2012).

**FIGURE 3:**
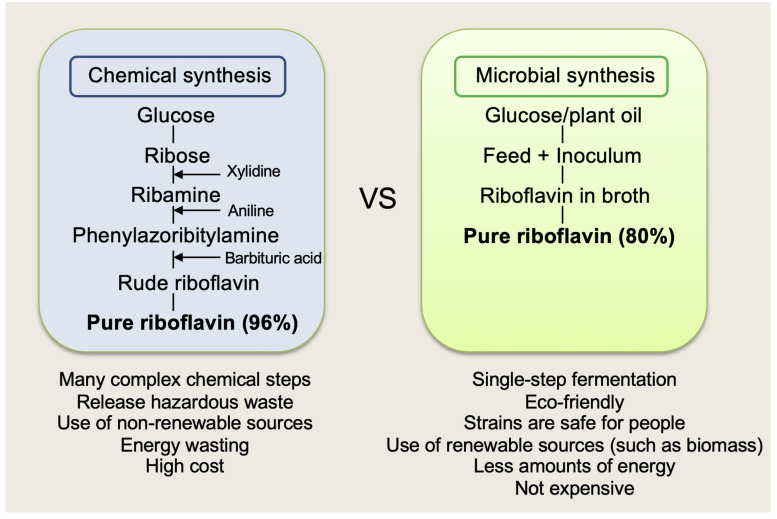
Chemical versus microbial riboflavin synthesis.

Commercial riboflavin production is currently based on industrial fermentation using overproducing strains of genetically engineered microorganisms. Chemical synthesis of vitamin B2 from ribose is being replaced by fermentation processes because of economic and environmental considerations of the latter (Figure 3). Besides the economic advantages, additional benefits of microbial synthesis include the use of renewable sources like sugar or plant oil, an environmental-friendly approach, and superior quality of the final product (Stahmann et al., 2000). For example, carbon dioxide emissions and the use of non-renewable resources are reduced by 80% and water emission by 66% each year. Examples of such bacterial overproducers are genetically engineered *B. subtilis*, *A. gossypii*, *E. ashbyii*, and *C. famata* riboflavin production strains. These strains are environmentally safe and often used in food and feed supplements industry. Today, strains of *A. gossypii* and *B. subtilis* are more preferable to riboflavin production because of the unstable fermentation process by *E. ashbyii* and *C. famata* (Stahmann et al., 2000). The largest worldwide producers of riboflavin production are BASF (Germany), DSM (formerly Roche) (Netherlands), Hubei Guangji Pharmaceuticals, and Shanghai Acebright Pharmaceuticals (formerly Desano) (China). More than 70% of the total riboflavin is used as feed additives for animal nutrition, known also as Vitamin B2 (80% grade); the other 30% is used in the food industry for specific nutritional purposes; for example, it is added to processed cereal-based foods, baby foods for infants and young children, and infant formulas and follow-on formulas; as a food colorant (riboflavin and riboflavin-50-phosphate), under numbers (i) E 101 and (ii) E-101; in medicine and veterinary science as a pharmacologically active substance for the treatment of diseases; and as an authorized colorant in cosmetic products (Commission Regulation (EU) 37/2010 of 22 December 2009,2010).

The synthetic industrial riboflavin process is described in detail by Stahmann et al. (2000). It begins with D-ribose reacting with 3,4-xylidine in methanol. This reaction step produces riboside. In this step, the industrial production of D-ribose can be obtained from glucose by *Bacillus* mutants lacking transketolase, a major enzyme of the pentose phosphate pathway (Competition Commission, 2001). The riboside formed is hydrogenated to give N-(3,4-dimethylphenyl)-D-1′-ribamine. This transient product is coupled with a phenyl diazonium halogenide, which produces an azo compound used in a cyclo-condensation with barbituric acid to give riboflavin. The main disadvantages of the procedure are: (1) it has a maximum yield of approximately 60% (from substrate), thus generating a lot of waste, (2) it requires organic solvents, and (3) it requires 25% more energy in comparison to the single-stage fermentation route.

The first commercial fermentations for riboflavin production were established using *C. acetobutylicum* (Leviton, 1946). The Merck company began riboflavin production by employing *A. gossypii* in 1974 (Stahmann et al., 2000).

Genetically improved *A. gossypii* strains have been used since 1990 for the industrial production of riboflavin by the chemical company BASF (Germany) (Aguiar et al., 2017). *A. gossypii* produces 40,000-fold more vitamin than it needs for its own growth. BASF ran a fermentation plant parallel with a chemical plant, but it was shut down in 1996. Later, this method replaced the seven-step chemical synthesis of riboflavin with one-step biosynthesis. In 2001, BASF and Takeda (Japan) formed a joint venture and optimized the biotechnological production of vitamin B2 using the fed-batch method with vegetable oils as carbon sources and soy/corn products as nitrogen sources (Igami and Sugaya, 2016). Further optimization on industrial riboflavin synthesis with *A. gossypii* led to riboflavin production greater than 20 g/L (Schwechheimer et al., 2016). The ADM (United States) improved the production of riboflavin using the yeast *C. famata* via aerobic fermentation, but later the plant was shut down due to the iron sensitivity of *C. famata* to the iron/steel equipment, which complicated the process (Abbas and Sibirny, 2011). However, there are some reports on iron-resistant industrial-scale strains that could produce up to 20 g/L (Heefner et al., 1992; Lim et al., 2001; Table 1). In 1998, the Swiss company Hoffmann-La Roche (henceforth Roche) manufactured 3000 t/annum riboflavin via chemical synthesis. In 2000, Roche replaced chemical production with microbial production using a genetically engineered roseoflavin-resistant *B. subtilis* RB50 strain (Perkins et al., 1999a,b; Hohmann et al., 2001) for over-production of riboflavin reaching concentrations greater than 10 g/L with glucose as a carbon source. Later, the method was overtaken by the company DSM (Hohmann et al., 2010). Constructed *B. subtilis* riboflavin-overproducing strains do not contain multiple copies of the *rib* operon, but have its strong phage-derived promoters and altered 5′-UTRs. The developed carbon-limited fed-batch method applied to industrial strains was available to recycle fermented biomass to obtain carbon and nitrogen supplies for a new fermentation cycle (Schwechheimer et al., 2016). In addition, DSM holds a patent for the invented process of riboflavin purification, particularly suitable for the removal of DNA associated with riboflavin crystals, a crucial step in food, feed, and pharmaceutical grade riboflavin production (Gloor, 2010). Hubei Guangji Pharmaceuticals (China) uses the fermentation method with *B. subtilis* proline-resistant strains that produce up to 26.5 g/L of riboflavin in 70 h (Schwechheimer et al., 2016).

Medium components before inoculum preparation and fermentation processes have to be sterilized separately by several groups (carbon sources, nitrogen sources, salts in water, and amino acids) to avoid Maillard reactions, in which products can become inhibitors for riboflavin production (Schwechheimer et al., 2016). Inoculation preparation includes use of low-concentration (2–10% v/v) inoculum broths containing young, undifferentiated mycelium devoid of spores and soporiferous sacs. Fermentation of riboflavin for *A. gossypii* is performed at the optimum temperature range of 26–30°C in fed-batch fermenters (100 m^3^), with the initial pH of the culture medium as approximately 6.5–7.5, in aerobic conditions for 6–8 days until the yield peaks (Stahmann et al., 2000; Schwechheimer et al., 2016). The process of fermentation by *B. subtilis* strain RB 50 is performed on carbon-limited fed-batch cultivations at a 35 m^3^ scale and has a short cycle time (48–56 h) (Bretzel et al., 1999). Riboflavin is synthesized and released into the culture broth at low growth rates under strict carbon-limited conditions of the feeding phase (Hohmann et al., 2001). Downstream processing begins with pasteurization of the broth to remove all viable cells of the production organism present in the final product. Due to low solubility of riboflavin in neutral aqueous solvents, part of the fermentation product accumulates as needle-like crystals in the broth, which can be easily separated from the biomass by centrifugation or filtration. Crystallization is completed in the crystallizer by evaporation of some water. Subsequent washing of crystals with hot diluted acids (hydrochloric or sulfuric acid) disrupts strain DNA. Further separation (via decantation, filtration, or centrifugation) followed by purification and drying (vacuum/spray drying) allows acquisition of a final product (powder/granulate) with a riboflavin content of up to 96% (feed-grade applications). An additional washing step and re-crystallization are used for human applications with 99% food-grade (Schwechheimer et al., 2016).

When comparing described industrial processes for two major riboflavin-producing strains, it is clear that the natural over-producing strain *A. gossypii* takes a long time due to the separation of growth and production phase, whereas the *B. subtilis* strain is fast growing and can produce riboflavin within 48 h, which makes it preferable for large-scale production (Paracchini et al., 2017). However, further studies on industrial riboflavin processes are needed to improve the main steps, such as fermentation conditions, purification, and availability to use recycled sources. Perhaps, usage of combined predictive models with advanced metabolic engineering techniques can help to optimize fermentation process and industrial strains for large-scale production of vitamin B2 in the future (Wu et al., 2007; Suzuki et al., 2011; Oraei et al., 2018).

## Discussion

Microorganisms isolated from various environmental sources are capable of synthesizing commercially valuable chemicals in many industries, including vitamins (particularly B-group as riboflavin and cobalamin), enzymes, and organic acids, which play a crucial role in humans and animals. In the last few years, the industrial production of riboflavin by major industrial strains—*B. subtilis* and *A. gossypii*—has achieved higher productivity, quality, and economic feasibility in white biotechnology. However, various parts of riboflavin biosynthesis in these strains remain unresolved. Particularly, the nature of phosphatase(s) in the riboflavin pathway is yet to be identified. Regulation of riboflavin accumulation and secretion into the culture medium, as well as the mechanism of action of several SNPs and other modifications on riboflavin production due to random mutagenesis still need to be elucidated and investigated. The reasons for the physiological role of riboflavin overproduction by *Candida* sp. under iron-restrictive conditions are still unknown. The mechanism underlying the metabolic regulation of carbon and nitrogen sources in riboflavin biosynthesis by responsible genes in yeasts and bacteria require investigation for better overproduction processes in further studies. The role and effect of various antimetabolites remain unknown, and this knowledge could be used to further improve riboflavin production. Moreover, further studies should be accelerated for the expansion of riboflavin production by manipulating the ability of novel recombinant strains via gene/protein engineering for the effective utilization of substrates and supplements, facilitating better methods for bioconversion with an economic and industrial perspective.

## Author Contributions

LA took the lead in writing the manuscript. LB and AP contributed to the design and implementation of the section describing improved riboflavin-producing microorganisms and in writing the manuscript. OS and LT contributed to the writing of the riboflavin manufacturing section. All authors provided critical feedback, helped shape the manuscript, and contributed to the final version of the manuscript.

## Conflict of Interest

The authors declare that the research was conducted in the absence of any commercial or financial relationships that could be construed as a potential conflict of interest.
